# Temporal and spatial dynamics of microbial communities and greenhouse gas flux responses to experimental flooding in riparian forest soils

**DOI:** 10.1093/femsec/fiaf109

**Published:** 2025-10-25

**Authors:** Kristel Reiss, Ülo Mander, Maarja Öpik, Siim-Kaarel Sepp, Kärt Kanger, Thomas Schindler, Kaido Soosaar, Mari Pihlatie, Klaus Butterbach-Bahl, Anuliina Putkinen, Ülo Niinemets, Mikk Espenberg

**Affiliations:** Institute of Ecology and Earth Sciences, University of Tartu, Vanemuise 46, Tartu 51003, Estonia; Institute of Ecology and Earth Sciences, University of Tartu, Vanemuise 46, Tartu 51003, Estonia; Institute of Ecology and Earth Sciences, University of Tartu, J. Liivi 2, Tartu 51003, Estonia; Institute of Ecology and Earth Sciences, University of Tartu, J. Liivi 2, Tartu 51003, Estonia; Netherlands Institute of Ecology (NIOO-KNAW), Droevendaalsesteeg 10, 6708 PB Wageningen, The Netherlands; Institute of Ecology and Earth Sciences, University of Tartu, Vanemuise 46, Tartu 51003, Estonia; Institute of Ecology and Earth Sciences, University of Tartu, Vanemuise 46, Tartu 51003, Estonia; Institute of Ecology and Earth Sciences, University of Tartu, Vanemuise 46, Tartu 51003, Estonia; Department of Agricultural Sciences, University of Helsinki, PO Box 56, Helsinki 00014, Finland; Institute for Atmospheric and Earth System Research, Faculty of Agriculture and Forestry, University of Helsinki, PO Box 56, Helsinki 00014, Finland; Department of Agroecology, Land-CRAFT, Center for Landscape Research in Sustainable Agricultural Futures, Aarhus University, Blichers Allé 20, 8830 Tjele, Denmark; Department of Agricultural Sciences, University of Helsinki, PO Box 56, Helsinki 00014, Finland; Institute for Atmospheric and Earth System Research, Faculty of Agriculture and Forestry, University of Helsinki, PO Box 56, Helsinki 00014, Finland; Chair of Crop Science and Plant Biology, Estonian University of Life Sciences, Kreutzwaldi 1, 51006 Tartu, Estonia; Institute of Ecology and Earth Sciences, University of Tartu, Vanemuise 46, Tartu 51003, Estonia

**Keywords:** arbuscular mycorrhizal fung, bacteria, fungi, methane, microtopography, nitrous oxide

## Abstract

Extreme rainfall and flooding are expected to increase in Northern subboreal habitats, altering soil hydrology and impacting greenhouse gas (GHG) fluxes by shifting redox potential and microbial communities as soils transition from aerobic to anaerobic conditions. This study examined the effects of a 2-week growing-season flash flood on bacterial, archaeal, and fungal communities and microbial processes driving CH_4_ and N_2_O fluxes in riparian alder (*Alnus incana*) forests. Flooding reduced soil nitrate accumulation as determined by quantitative polymerase chain reaction and promoted dinitrogen-fixing, *nifH* gene-carrying bacteria like *Geomonas*. Sequencing data showed that anaerobic bacteria (*Oleiharenicola, Pelotalea*) increased during the flood, while N_2_O emissions declined, indicating a shift towards complete denitrification to N_2_. However, drier patches within the flooded area emitted N_2_O, suggesting nitrification or incomplete denitrification. A diverse arbuscular mycorrhizal community was detected, including genera *Acaulospora, Archaeospora, Claroideoglomus, Diversispora*, and *Paraglomus*. Flooding increased the abundance of the fungal genera *Naucoria, Russula*, and *Tomentella* and the family *Thelephoraceae*, which symbiotically support alder trees in nitrogen uptake and carbon sequestration. Microtopographic differences of 0.3–0.7 m created spatial variability in GHG emissions during flooding, with some waterlogged areas emitting CH_4_, while others enhanced CH_4_ oxidation (determined by FAPROTAX) and promoted nitrification-driven N_2_O emissions in drier, elevated zones. We conclude that flash flooding during the active growing season significantly affects nitrogen-fixing and nitrifying microbes and alters symbiotic fungal community composition, creating spatial variability in GHG emissions.

## Teaser

Extreme rainfall and flooding are increasing in Northern forests, changing soil water levels and microbial communities, which affects how greenhouse gases.

## Introduction

The International Panel on Climate Change (IPCC) reported that atmospheric carbon dioxide (CO_2_) levels are the highest in 2 million years, with methane (CH_4_) and nitrous oxide (N_2_O) concentrations also significantly rising (Reed et al. 2011, IPCC 2021). These increases are associated with climate warming and a rising frequency of extreme climate events, such as intense rainfall and floods (IPCC 2021). Such extremes alter soil hydrological regimes (Schindler et al. 2020, Furtak and Wolińska 2023) and impact microbial communities, including their diversity and function (Unger et al. 2009, Hutchins et al. 2019). The changes in soil prokaryotic and fungal abundances and communities are often associated with shifts in N_2_O (Zhang et al. 2021a, Espenberg et al. 2018) and CH_4_ (Wang et al. 2023, Yang et al. 2024) fluxes.

Riparian ecosystems, which act as periodically flooded buffer zones (Naiman et al. 2010), can help mitigate these climate-driven changes. They intercept the excess nutrients transported via flood and soil runoff (Peter et al. 2012, Lyu et al. 2021), bind nutrients with organic matter (OM) (Lidman et al. 2017), and retain pollutants (Riis et al. 2020). Their nutrient-binding capacity depends on factors, such as soil type (Pinay et al. 1995), topography (Adhikari et al. 2018), buffer width (Lyu et al. 2021, Graziano et al. 2022, Mander et al. 1997), vegetation (Petersen et al. 2020, Tolkkinen et al. 2020), hydrology (Tolkkinen et al. 2020), and groundwater level fluctuations (Hefting et al. 2004). Riparian forests also provide aerobic and anaerobic microbial habitats that drive soil biogeochemical processes involved in the production and consumption of greenhouse gases (GHGs) such as CH_4_ and N_2_O (Annala et al. 2022). Additionally, the impact of flooding on subboreal vegetation depends significantly on its duration and timing (Sarneel et al. 2019). In riparian areas, flooding typically occurs in spring and autumn when vegetation is mainly dormant and exhibits reduced physiological activity. However, intense rainfall can also cause flooding during the peak growing season, potentially resulting in severe damage to vegetation (Van Eck et al. 2006). European riparian areas total ~91 144 km^2^, with northern countries like Finland and Estonia having the highest relative cover (Clerici et al. 2013). In North-Eastern Europe, grey alder (*Alnus incana* (L.) Moench) is common in riparian zones (Lõhmus et al. 1996, Aosaar et al. 2012, Uri et al. 2014) due to its adaptation to high soil moisture conditions (Johansson 1999).

Studies in riparian areas show that flooding and higher groundwater levels can increase CH_4_ emissions from grey alder (*A. incana*) stands (Soosaar et al. 2011, Mander et al. 2015). Net CH_4_ release occurs in soils where methanogenic archaea, the final recipients of substrates originating from OM fermentation, outcompete methanotrophs in their activity (Guerrero-Cruz et al. 2021). Emissions occur mainly through diffusion, bubbles, and mass flow through plant aerenchyma (Le Mer and Roger 2001). Oxidation of CH_4_ occurs primarily by aerobic methanotrophs (Le Mer and Roger 2001) but can also take place anaerobically (Boetius et al. 2000) via nitrite-dependent anaerobic methane oxidation (n-damo) by bacteria from the NC10 phylum (Ettwig et al. 2009), or via the archaeal domain (e.g. *Methanoperedenaceae*), oxidizing CH_4_ by reverse methanogenesis (Cui et al. 2015). CH_4_ emissions vary seasonally, with warmer temperatures promoting methanogenic activity (Li et al. 2023). Riparian areas are traditionally known as CH_4_ sinks, but the increased frequency of long-term and short-term flooding may alter them from sinks to CH_4_ sources in periods of prolonged, flooding-induced soil anaerobiosis (Jacinthe 2015). Additionally, higher precipitation and, thus, soil moisture reduce soil’s ability to oxidize CH_4_, leading to increased emissions (Guo et al. 2023).

Flooding can also increase N_2_O emissions, with higher emissions observed after short-term flooding (Jacinthe et al. 2012) or immediately after rewetting (Harris et al. 2021). N_2_O is mainly a by-product of nitrification or denitrification (Hallin et al. 2018) but may also be emitted through dissimilatory NO_3_^−^ reduction to ammonia (NH_3_^+^) (DNRA) under certain conditions (Rütting et al. 2011, Mania et al. 2014). In riparian zones, the importance of denitrification has been highlighted (Mander et al. 2014). Additionally, complete nitrifiers known as comammox can oxidize NH_3_^+^, but the potential for N_2_O production remains unclear (Koch et al. 2019). In organic-rich soils, N_2_O emissions are influenced by temperature, soil moisture, NO_3_^−^ concentration (Pärn et al. 2018), and vegetation (Hernandez and Mitsch 2006). In grey alder forests, biological dinitrogen (N_2_) fixation is another key process, converting N_2_ to NH_4_^+^, accessible to plants. It has been shown that soil water content is an important factor in N_2_ fixation, and decreased soil water content can decrease biological N_2_ fixation (Whiteley and Gonzalez 2016, Zhang et al. 2020). Grey alder forms a symbiotic relationship with N-fixing bacteria from the genus *Frankia* (Franche et al. 2009, Sellstedt and Richau 2013). Increased water availability has also been shown to result in increased N_2_ fixation rates by a diverse group of free-living N_2_-fixers (Reed et al. 2011).

In addition to the prokaryotic microbes, changes in the fungal community can significantly affect ecosystem carbon (C) cycling and storage (Orwin et al. 2011). Fungi are essential in several ecosystem processes, including C sequestration (Clemmensen et al. 2013), and decomposition of litter (Kubartová et al. 2009) and organic C (Treseder and Lennon 2015). Most plants form symbiotic relationships with fungi, which transfer nutrients like nitrogen (N) and phosphorus (P) from soil to plants (van Der Heijden et al. 2015) in exchange for carbohydrates and fatty acids (Diagne et al. 2020). Fungi also play an essential role in the N cycle (Gui et al. 2021, Huaisong et al. 2024). Arbuscular mycorrhizal fungi (AMF) can influence the abundance of genes involved in soil N cycle, indirectly affecting N_2_O emissions (Okiobe et al. 2022, Qiu et al. 2022). In addition, N_2_O can be produced through fungal denitrification, a process in which fungi reduce nitrite (NO_2_^−^) to nitrous oxide N_2_O (Shoun et al. 2012). The phylum *Ascomycota* includes fungi that produce N_2_O (Mothapo et al. 2015), such as certain species of *Fusarium* (Lin et al. 2024).

Riparian areas are complex ecosystems, where aerobic and anaerobic microbial C and N processes can cooccur, making it crucial to study both together and link them to temporal changes in soil redox potentials (Espenberg et al. 2024). While many studies have explored the effects of environmental factors such as rainfall or temperature on GHG emissions, the impact of ecological disturbances, such as flooding on microbial processes and community structures requires further analysis (Cavicchioli et al. 2019). There are particularly few studies on the impact of growing season flooding on riparian microbial communities and associated changes in GHG emissions. This study investigated how short-term flooding during the growing season influences the soil microbiome and the importance of flooding periods in shaping soil microbial N and C cycles and associated GHG production, oxidation, and emission processes in a riparian alder forest. It focused on how soil bacteria, fungi, and AMF communities relate to physico-chemical characteristics and soil CH_4_ and N_2_O emissions during short-term flooding.

The study hypothesized that: (1) experimental flooding during the plant growing season decreases bacterial and fungal diversity; (2) flooding-dependent changes in the microbiome are responsible for decreases in N_2_O emissions and increases in CH_4_ emissions after flooding; and (3) rapidly changing soil moisture content and induced soil anaerobiosis mainly affects nitrifying microbial communities, including microbes mediating the complete ammonia oxidation (comammox) process.

## Materials and methods

### Site description, experimental set-up, and soil sampling

The experimental site (58° 17′ N; 27° 17′ E, 36 m asl) is in a riparian forest near the Kalli River in Agali, Tartu County, Estonia (Fig. S1). The area is situated on gley soil, and the thickness of the raw humus horizon at the study site ranges from 15 to 20 cm (Mander et al. 2022). The site is a former agricultural land dominated by a 40-year-old grey alder [*A. incana* (L.) Moench] forest, and the understory includes bird cherry (*Prunus padus* L.), meadowsweet [*Filipendula ulmaria* (L.) Maxim.], and raspberry (*Rubus idaeus* L.) The experimental site was divided into a control plot (CP) and an experimental plot, i.e. the flooded plot (FP). The FP measured ca 40 m × 40 m, and the CP was ca 20 m × 20 m, separated by a natural 1-m dyke. To mimic an intensive rainfall creating a runoff, each day during the flooding (14 days), 55–70 m^3^ of water was transported to the FP by a fire truck.

The experiment consisted of four intensive measuring periods and, and each period consisted of two sampling campaigns, except the final sampling period (POSTPOST), which consisted of one campaign (altogether seven sampling campaigns). Soil samples were collected during the preexperimental period (PRE) on 25 or 26 July and 7 August 2017, during the artificial flooding period (EXP) on 16 and 21 August 2017 (the 8th and 13th day of the flood), and during the postexperimental period (POST) on 11 or 12 September and 7 November 2017 (21 or 22 days after the flooding and 78 days after the flooding, respectively). The final soil samples were collected the following year, on 15 August 2018 (POSTPOST).

During each sampling campaign, 12 samples were collected: 8 from the FP and 4 from the CP. We designed the study so that the flooded and CPs were as close together as possible to ensure comparability, while ensuring that floodwater did not affect the CP. Sampling locations within plots were randomly selected. The soil samples were taken at a depth of 0–10 cm with a spade from the vicinity of the grey alder trees. Samples were collected following a random sampling strategy, where each composite sample was formed by pooling three subsamples from the same soil layer. A total of 84 composite samples were collected. The samples were stored in a refrigerator (+4°C) for chemical and in a freezer (−20°C) for microbiological analyses. A more detailed description of the site, experimental setup, and sampling procedures is available in Schindler et al. (2020).

### Physiochemical analyses and GHG emission analyses

Soil temperature, water content and soil moisture were measured during the experiment at 0–10 cm depth. Soil pH_KCl_, total nitrogen (N%), NO_3_^−^, NH_4_^+^, total P, potassium (K), calcium (Ca), magnesium (Mg), and OM contents were measured using the standard protocol (APHA-AWWA-WEF 2005). Soil N_2_O and CH_4_ fluxes were measured 12 times daily with 12 automated chambers, each covering 0.16 m^2^ forest floor and placed near representative grey alder trees. A Picarro G2508 (Picarro Inc., USA) gas analyser with cavity ring-down spectroscopy technology was used to monitor GHG gas concentrations. A detailed description of the physico-chemical analyses and GHG measurements can be found in Schindler et al. (2020).

### Soil DNA extraction

The PowerSoil® DNA Isolation Kit (Qiagen, USA) was used to extract DNA from the soil samples (0.25 g), following the manufacturer’s protocol. The samples were homogenized for 20 s at 5000 r/m using a Precellys® 24 homogenizer (Bertin Technologies SAS, France). The quantity and quality of the DNA were assessed using a spectrophotometer Infinite M200 (Tecan Trading AG, Switzerland). The extracted DNA was stored at −20°C until further analysis.

### Quantification of gene copies using quantitative polymerase chain reaction

Functional estimates of microbial biogeochemical processes were based on quantifying established functional marker genes using real-time quantitative polymerase chain reaction (qPCR). The qPCR analyses were performed using a Rotor-Gene Q thermocycler (Qiagen, USA). The 10 μl reaction mixture for amplification contained 5 μl of Maxima SYBR Green Master Mix (Thermo Fisher Scientific Inc., USA), optimized forward and reverse primers, 1 μl of isolated sample DNA, and sterile distilled water. Measurements with qPCR were performed in triplicate for each sample, and negative controls were included for each measurement.

Rotor-Gene Series Software v 2.0.2 was used to analyse the amplification and melting curves of the samples, and LinReg PCR v 2020.0 was used to consider the amplification efficiencies of the samples. Gene copy numbers were calculated using the corresponding standard curve for each marker gene, and gene abundances were expressed as the number of gene copies per gram of dry matter (copies/gDM). The qPCR standard curves were constructed using plasmids containing cloned gene fragments (Eurofins Genomics, Germany). Standard curves were then generated from serial dilutions of these plasmid DNA stock solutions, ranging from 10^9^ to 25 copies per 10 μl of qPCR reaction mixture, depending on the target gene. The exact dilution series used are reported in Table S1.

Functional genes related to the N cycle were quantified, using primers nirSCd3 and nirSR3cd (Kandeler et al. 2006) for *nirS*, nirK876 and nirK1040 (Hallin and Lindgren 1999) for *nirK*, nosZ2F and nosZ2R (Henry et al. 2006) for *nosZ* clade I (*nosZI*), nosZ-II-F and nosZ-II-R (Jones et al. 2013) for *nosZ* clade II (*nosZII*) (prokaryotic denitrification), FnirK-F3 and FnirK-R2 (Chen et al. 2016) for fungal *nirK* (fungal denitrification), amoA-1F and amoA-2R (Rotthauwe et al. 1997) for bacterial and CrenamoA 23F and CrenamoA 616R (Tourna et al. 2008) for archaeal *amoA* (nitrification), comamoA AF and comamoA SR (Wang et al. 2018) for COMAMMOX *amoA* (complete ammonia oxidation), 6RF and 6R (Takeuchi 2006) for *nrfA* (DNRA), and Ueda19F and Ueda407R (Ueda et al. 1995) for *nifH* (nitrogen fixation). For CH_4_ cycle processes the following primers were used: mcrA-F and mcrA-R (Espenberg et al. 2016) for *mcrA* (methanogenesis), A189F and Mb661R for *pmoA* (methanotrophy), and pq2F and pq2R for (Ettwig et al. 2009) n-damo-specific 16S rRNA (nitrite/nitrate-dependent anaerobic CH_4_ oxidation) genes. All primer sets for target genes, amplification efficiencies, optimized primer concentrations, and optimized qPCR programs are listed in Table S1.

### Preparation of DNA libraries, sequencing, and data processing

We used a soil metabarcoding approach to characterize the bacterial, fungal, and more specifically, the AMF community. Total environmental DNA was extracted from bulk soil samples (not specifically picked from roots) as stated above, and specific marker genes were subsequently amplified from the DNA extracts. Sequencing was performed using bacterial 16S rRNA gene primers 515F and 806rB (Caporaso et al. 2011), general fungal-specific Internal Transcribed Spacer (ITS) region primers fITS7o, fITS7, and ITS4 (White et al. 1990, Ihrmark et al. 2012, Kohout et al. 2014), and AMF-specific SSU rRNA gene primers WANDA and AML2 (Lee et al. 2008, Dumbrell et al. 2011). Sequencing was conducted on the Illumina MiSeq platform with 2 × 250 bp and 2 × 300 bp chemistry for bacteria and all fungi or AMF, respectively (Illumina, California, USA). Amplicon-based polymerase chain reaction (PCR) and sequencing were carried out by Asper Biogene OÜ (Tartu, Estonia). Although the universal prokaryotic 16S rRNA primers 515F and 806rB capture some of the archaeal community, they have been shown to severely underestimate its diversity (Bahram et al. 2019). Hence, we did not include archaeal sequences in downstream analysis.

The DADA2 package (Callahan et al. 2016) was used to aggregate demultiplexed raw sequencing reads into exact amplicon sequence variants (ASV). Primer sequences were removed from bacterial and fungal fragments using the cutadapt program (Martin 2011). Due to ambiguous nucleotide bases (Ns) and very short sequences, filtering was repeated after primer removal using the *filterAndTrim()* function from the DADA2 package, with MaxN = 0, truncQ = 2, minLen = 50, and rm.phix = TRUE. After filtering, the sequences were assembled using the *makeSequenceTable()* function, and taxonomy was added using the *assignTaxonomy()* function from the DADA2 package. The SILVA v138.2 database (Quast et al. 2013) was used for bacterial taxonomy assignment. The SILVA database taxonomy was formatted to fasta files (Callahan 2024) and used in the DADA2 workflow. The UNITE database (Kõljalg et al. 2020) was used for fungal taxonomy assignment. The MaarjAM database (Öpik et al. 2010) was used for AMF taxonomy assignment (identification to match against virtual taxa, VT).

The software ‘R version 4.3.2’ and ‘RStudio 2022.12.0’ were used for data processing and bioinformatic analyses. Bacterial ASV, fungal ASV, and AMF VT data, samples, and taxonomy tables were used to create *phyloseq* objects with the Phyloseq package (McMurdie and Holmes 2013). In the *phyloseq* object, the relative abundance was calculated using the *transform_sample_counts()* function from the Phyloseq package. The Phyloseq functions *estimate_richness()* and *plot_richness()* were used to estimate bacterial and fungal diversity (McMurdie and Holmes 2013). The Shannon (H’) index was calculated to assess α-diversity (Xia and Sun 2023). The Shannon (H’) index is calculated using the following formula: ${\mathrm{H^{\prime}}} = - \mathop \sum \limits_{i = 1}^S {{p}_i}{\mathrm{ln}}( {{{p}_i}} ),$ where *p_i_* is the proportional abundance of genera *i, S* is the number of unique genera, and ln is the natural logarithm (Oksanen 2024).

In addition, bacterial ASV data were functionally annotated using the FAPROTAX database (Louca et al. 2016) to estimate the potential biogeochemical functions of the bacterial community based on the relative abundances of ASV sequences.

Total number of raw reads has been added to the Table S9. Sequence data from this study have been deposited in the European Nucleotide Archive with the primary accession code PRJEB86668.

### Spatial analysis

The software ‘ArcGIS Pro 3.3.3’ was used to analyse and map the temporal and spatial changes of environmental characteristics and soil microbial diversity. Using the *Minimum Bounding Geometry* tool, a polygon for interpolation was created from sample area points. The geometry type was set to envelope. The sample area polygon was extended 5 m outward from the points to visualize the edge effects during interpolation. A 1-m resolution geoTIFF from the Estonian Land and Spatial Development Board (Land and Spatial Development Board 2025) was used for surface elevation data (map sheet 54 693, at a 1:10 000 scale). Elevation values were obtained for each sampling point using the *Extract Values to Points* tool. The surface elevation model was used to create a Local Scene, which displays 3D maps in ArcGIS Pro. A five-fold vertical exaggeration was applied to the terrain to show the area’s terrain on the map.

The inverse distance weighted (IDW) interpolation method was used for interpolation. The Spatial Analyst toolset applied the IDW interpolation method, and the power was set to 2. Power is the distance exponent and controls the significance of surrounding points (Esri 2025). Average values for each period were used for interpolating soil emissions, soil moisture, sequencing data (methanotrophy, NO_3_^−^ reduction, and N_2_ fixation), and qPCR data (marker gene abundances).

### Statistical analyses

The software ‘R version 4.3.2’ (R Core Team 2023) and ‘RStudio 2022.12.0’ were used for statistical analysis. Friedman *post hoc* pairwise Wilcoxon test with *pairwise_wilcox_test()* function from the rstatix package (Kassambara 2023a) was used to see which specific pairs of experimental periods (PRE, EXP, POST, and POSTPOST) differed in marker gene abundances. Also, two-sample *t*-tests were conducted to evaluate the effect of flooding on marker gene abundances within each experimental period. Log-transformed abundances were subset by period, and the Welch two-sample *t*-test was applied to compare control and treatment groups. The analysis was performed using the *t.test()* function from the *stats* package in R (R Core Team 2023), and *P*-values below .05 were considered statistically significant.

In addition to that, the Kruskal–Wallis test was used to determine the significant differences between each marker gene abundance in different experimental periods and sample areas. Pairwise comparisons for marker genes with a significant result (*P* < .05) in the Kruskal–Wallis test were conducted using the Wilcoxon rank-sum test. Functions *kruskal.test()* and *pairwise.wilcox.test()* from the *stats* package (R Core Team 2023) was used, and adjusted *P*-values below .05 were considered statistically significant. Benjamini–Hochberg corrected adjusted *P-*values (*P* < .05) were used.

Two-sample *t*-tests were conducted to evaluate the effect of flooding on marker gene abundances within each experimental period. Log-transformed abundances were subset by period, and the Welch two-sample *t*-test was applied to compare control and treatment groups. The analysis was performed using the *t.test()* function from the *stats* package in R (R Core Team 2023), and *P*-values below .05 were considered statistically significant.

The Wilcoxon test was applied to evaluate statistically significant relationships between diversity indices The *stat_compare_means()* function from the ggpubr package (Kassambara 2023b) was used to assess the changes in diversity indices during the experimental period. Significance of changes in bacterial and fungal genera and AMF family relative abundance were tested with the Wilcoxon test wilcox.test() function; Benjamini–Hochberg corrected adjusted *P*-values (*P* < .05) were added with *p.adjust()* function from stats package (R Core Team 2023). The average diversity indices and genera abundance of the PRE and EXP, EXP, and POST, and PRE and POST were compared in both sampling areas (FP and CP).

Multivariate linear models were used to assess soil environmental factors and emissions across different time periods and plots. Model significance was evaluated using 9999 permutations via the *anova()* function from the mvabund package (Wang et al. 2022b). Spearman rank correlation coefficients were used to assess the relationships between environmental factors, diversity, and target gene abundances in the different experimental periods on the FP. The *rcorr()* function from the Hmisc package (Harrell Jr. 2019) was used to calculate the Spearman correlations and the significance (*P* < .05) of the relationship.

The Mantel test was used to show a significant relationship between physico-chemical characteristics, marker gene abundances (qPCR), functional processes and diversity indices (sequencing data) with soil N_2_O and CH_4_ emissions in the different experimental periods on the FP. Matrices with environmental and emission data mean values were created for each experimental period × sample combination. A point matrix was created from the longitude and latitude data of the sample points. The function *mantel.partial()* from the vegan package (Oksanen et al. 2022) was used for the Mantel statistic representing matrix correlation between three dissimilarity matrices. Spearman correlation method with 999 permutations was used.

Redundancy analysis (RDA) and principal components analysis (PCA) were used to explore relationships between microbial communities (bacteria, fungi, and AMF), environmental variables and soil fluxes. The missMDA package (Josse and Husson 2016), which imputes missing values, was used to fill in the environmental data table for RDA analysis. The function *estim_ncpPCA()* was used to estimate the number of dimensions to use, and the *imputePCA()* function was used to fill in the data gaps with the previously estimated number of dimensions. RDA and PCA plots were created with the *tax_transform(), ord_calc()*, and *ord_plot()* functions from the microViz package (Barnett 2024) and 15 most abundant genera were plotted. Microbial taxa relative abundances were transformed using the centred log-ratio transformation (‘clr’) prior to applying PCA and RDA. The RDA and PCA microbial data were aggregated with *tax_agg()* function, displayed at the genus level (family level for AMF), and visualized with the ggplot2 package (Wickham 2016). The RDA graphs were divided into three separate plots to enhance readability and clarity. PCA graphs are presented in Fig. S2. Permutation-based multivariate analysis of variance (PERMANOVA) was used to assess the effect of treatment, experiment, and their interaction on the bacterial, fungal, and AMF communities, using the vegan package *adonis2* (Oksanen et al. 2022) with Bray–Curtis dissimilarity and 999 permutations.

Microbial community composition was analyzed using nonmetric multidimensional scaling (NMDS) based on Bray–Curtis dissimilarities. Prior to ordination, relative abundance was calculated and low-abundance genera were filtered to retain the 15 most abundant taxa. NMDS was performed using the *ordinate()* and *plot_ordination()* functions from phyloseq package (McMurdie and Holmes 2013). NDMS figures are added to the Supplementary material (Figs S8–S10).

## Results

### Soil CH_4_ and N_2_O fluxes

**Figure 1.**
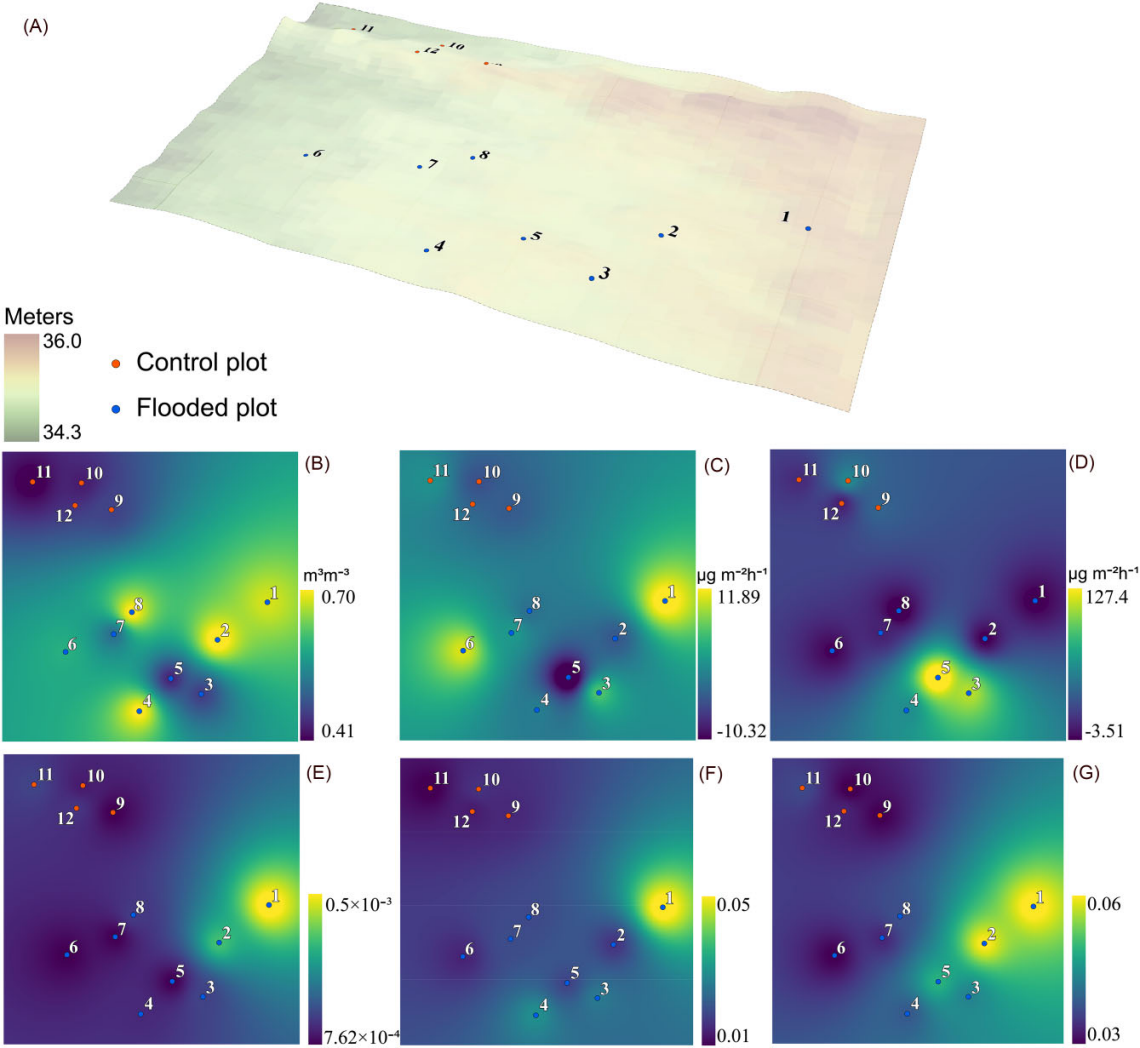
Sampling area elevation (A), soil moisture (B), soil CH_4_ fluxes (C), N_2_O fluxes (D), methanotrophy (E), NO_3_^−^ reduction (F), and N_2_ fixation (G) during the EXP period. CP sampling points (9-12) are shown in red, and FP sampling points (1-8) are shown in blue. The relative abundance of functionally annotated taxa from the sequencing data (FAPROTAX) was used for interpolation (E–G).

The sampling area elevation range is shown in Fig. 1(A). Soil moisture was higher in the FP during the EXP period than in the CP, but points 3 and 5 in the FP had lower soil moisture than other FP sampling points (Fig. 1B). Flooding had no significant effect on soil N_2_O emissions (Schindler et al. 2020), but interpolation results showed that the points with lower soil moisture in the FP (points 3 and 5) had the highest N_2_O emissions (Fig. 1D). These points are located at higher elevations than the surrounding area. Soil CH_4_ emissions were significantly higher in the FP during the EXP (Schindler et al. 2020). However, the interpolation results indicate that there are points in the FP that act as both CH_4_ sinks and sources (Fig. 1C).

Functional annotation of prokaryotic taxa (FAPROTAX) revealed that genetic potential for methanotrophy was highest at points 1 and 2 in the FP (Fig. 1E). The highest proportion of NO_3_^−^ reducers was also observed at point 1 (Fig. 1F). On average, FPs hosted a higher proportion of NO_3_^−^ reducers and N_2_ fixers (Fig. 1G).

### Bacterial relative abundance and diversity

**Figure 2.**
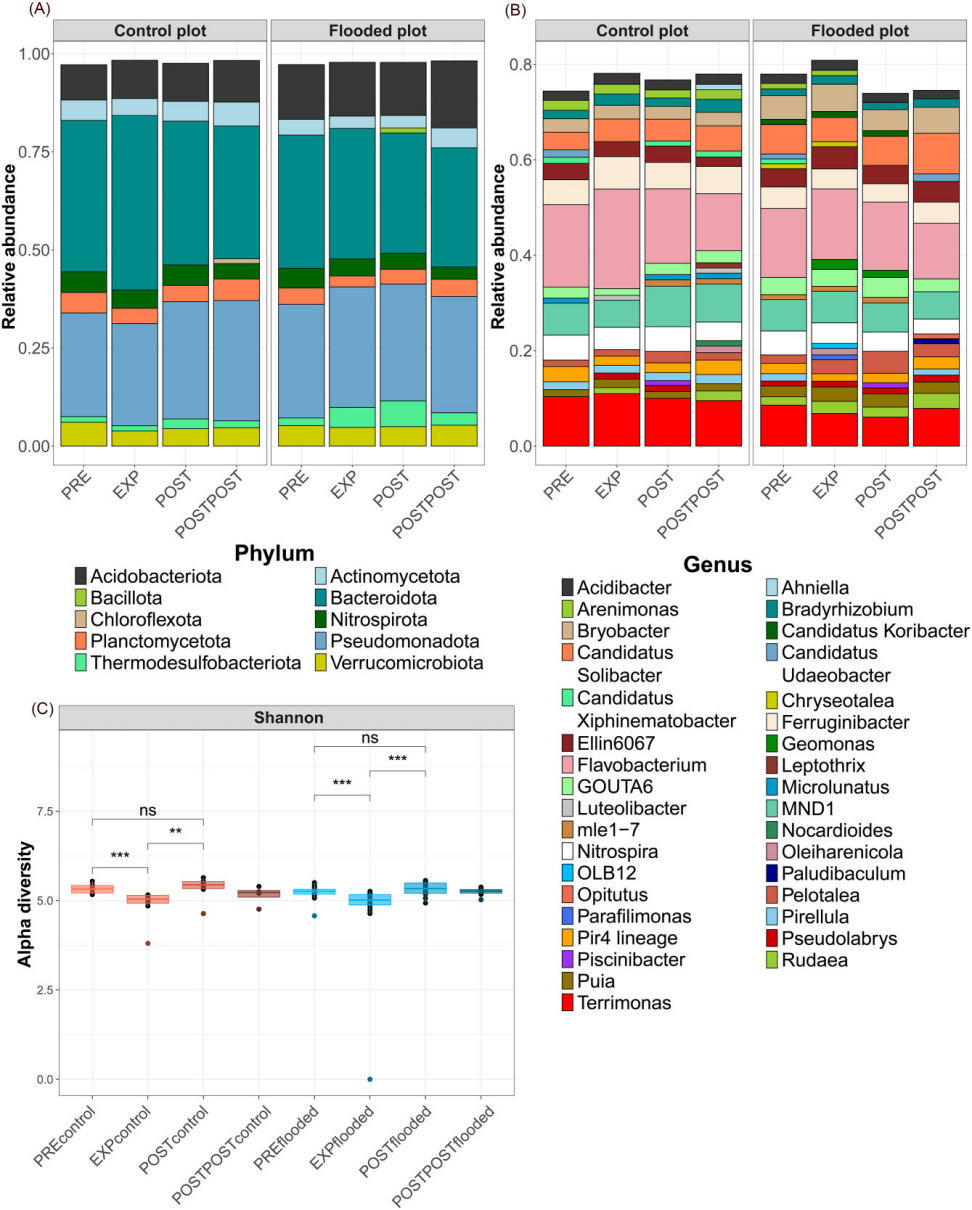
Relative abundances of bacteria at the phylum (A) and genus (B) levels in the CP and the FPs during different experiment periods in the riparian *A. incana* forest. Taxonomic classification of sequencing data was performed using the SILVA v138.2 database (Quast et al. 2013). Panel C shows changes in the bacterial Shannon index during the experiment. Red bars represent CP and blue bars represent FP. The asterisk (*) indicates a statistically significant difference between the experiment periods (*P* < .05 = ‘*’, *P* < .01 = ‘**’, and *P* < .001 = ‘***’). The flooding consisted of a 14-day continuous soil inundation during the growing season. Abbreviations: preflood period (PRE), flooding period (EXP), postflood period (POST), and a year after the flooding (POSTPOST).

Based on sequencing data, the bacteria in the study area were classified into 47 phyla and 316 different genera. The most abundant phyla were *Bacteroidota, Pseudomonadota, Acidobacteriota, Verrucomicrobiota*, and *Nitrospirota* (Fig. 2A). The most abundant genera were *Flavobacterium, Terrimonas, MND1*, Candidatus *Solibacter*, and *Ferruginibacter* (Fig. 2B).

Within study plots, Shannon index (H’) of bacteria showed statistically significant differences between the preflood period (PRE) and flooding period (EXP) periods (CP: *P* < .001, FP: *P* < .001) and between the EXP and postflood period (POST) periods (CP: *P* < .01, FP: *P* < .001) in both plots (Fig. 2C), with the index decreasing during the EXP period and increasing again during the POST period. Also, PERMANOVA (Table S6) indicated that the treatment had a small, yet statistically significant effect (*R*^2^ = 0.059, *P* < .001) on the bacterial community composition (Fig. S2). Experimental periods also showed a statistically significant effect (*P* < .01) on bacterial community composition (*R*^2^ = 0.052). Also, NMDS results showed that the bacterial community in the FP was more variable, with points distributed over a wider area that also encompassed the CP (Fig. S8).

**Figure 3.**
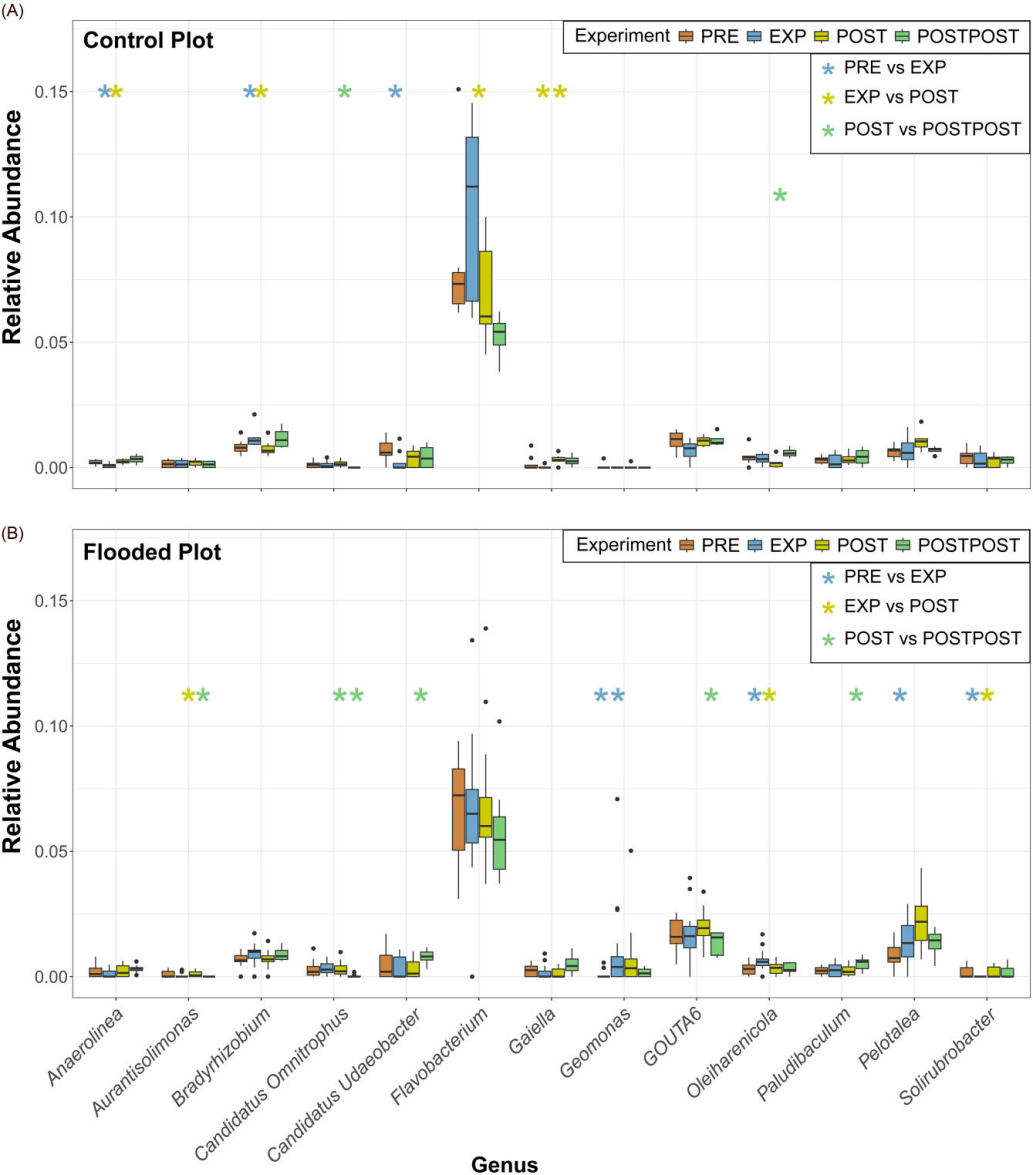
Changes in the relative abundance of bacterial genera in the CP (A) and FP (B), based on sequencing data. The Wilcoxon rank-sum test determined statistically significant differences between periods (PRE vs. EXP, EXP vs. POST, and POST vs. POSTPOST). Asterisks (*) indicate statistical significance (*—*P* < .05, **—*P* < .01, and ***—*P* < .001), with blue, yellow, and green denoting changes between PRE-EXP, EXP-POST, and POST-POSTPOST, respectively. Abbreviations: preflood period (PRE), flooding period (EXP), postflood period (POST), and a year after the flooding (POSTPOST).

The relative abundance of the genus *Flavobacterium* decreased in both study areas after the EXP period. However, the decrease was significant only in CP (*P* < .05) (Fig. 3A). The relative abundance of *Bradyrhizobium* increased significantly during EXP period (*P* < .05) and decreased in POST period (*P* < .05) in the CP. In contrast, the relative abundance of genus *Gaiella* increased (*P* < .01) after the EXP period in the CP. The relative abundances of *Bradyrhizobium* and *Gaiella* did not change significantly in the FP.

The relative abundance of Candidatus *Omnitrophus* decreased substantially in both study areas (CT *P* < .05; FP *P* < .01). The relative abundance of Candidatus *Udaeobacter* decreased significantly during the EXP period in the CP (*P* < .05), and only significant change in the FP occurred a year later when the relative abundance increased (*P* < .05).

In the FP, the relative abundances of *Geomonas, Oleiharenicola*, and *Pelotalea* (Fig. 3B) increased significantly (respectively, *P* < .01, *P* < .05, and *P* < .05) during the flood. The relative abundance of *Solirubrobacter* decreased significantly during the flood (*P* < .05) and increased after the flood (*P* < .05) in the FP. The relative abundance of *Aurantisolimonas* also increased significantly after the flood (*P* < .05) and decreased a year later (*P* < .05). The relative abundance of *GOUTA6* decreased (*P* < .05), while *Paludibaculum* increased significantly (*P* < .05) a year later (POSTPOST period) in the FP.

No significant changes in the relative abundances of *Geomonas, Pelotalea, Aurantisolimonas, GOUTA6*, and *Paludibaculum* were observed in the CP. Additionally, the relative abundances of ammonia-oxidizing bacteria (AOB) from the *MND1* and *Ellin6067* genera (Fig. S3) exhibited changes during the flood; however, the observed increases in abundance were not statistically significant.

### Fungal relative abundance and diversity

**Figure 4.**
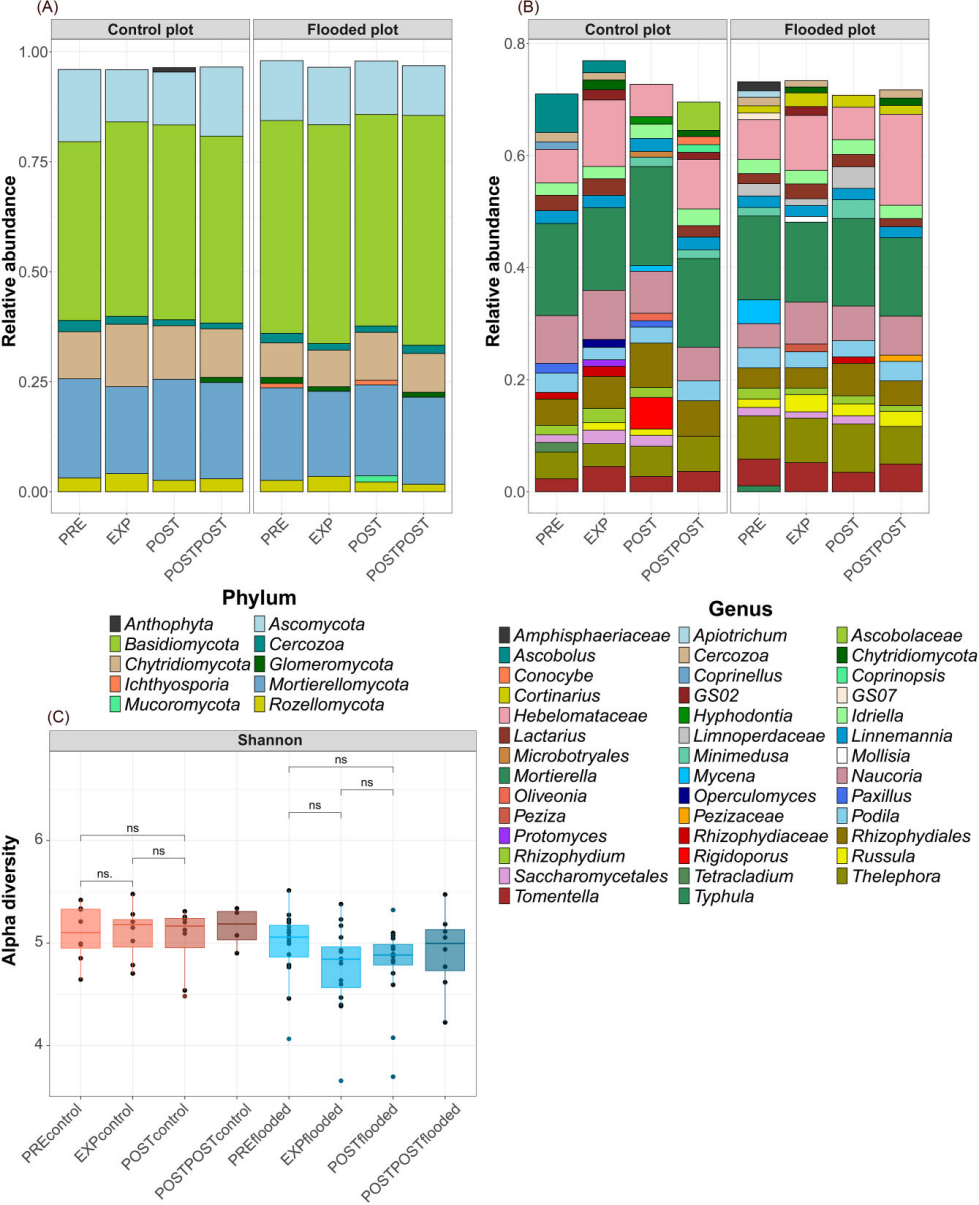
Relative abundances of fungi at the phylum (A) and genus (or family) (B) levels in the CPs and the FPs during different experiment periods, based on sequencing data. Panel C shows changes in the fungal Shannon diversity index during the experiment. Red bars represent CP, and blue bars represent FP. The asterisk (*) indicates a statistically significant difference between the experiment periods (*P* < .05 = ‘*’, *P* < .01 = ‘**’, and *P* < .001 = ‘***’). Abbreviations: preflood period (PRE), flooding period (EXP), postflood period (POST), and a year after the flooding (POSTPOST).

The proportions of fungal phyla were generally similar between the sampling areas and experimental periods. Based on sequencing data, 34 phyla were identified in the sampling areas, all represented in the FP and 32 in the CP. PCA and (Fig. S2B) NMDS analysis (Fig. S9) revealed overlapping fungal communities in the flooded and CPs, with the dominant genera occurring in both sample area (Fig. S8). Consistently, PERMANOVA (Table S7) and the Shannon index (H’; Fig. 4C) indicated no significant differences in fungal diversity between treatments.

The most abundant fungal phyla were *Basidiobolomycota, Mortierellomycota, Ascomycota, Chytridiomycota*, and *Rozellomycota* (Fig. 4A). 714 fungal genera were identified in the sampling area, with 623 in the FP and 542 in the CP. The most abundant fungal genera in both areas were *Mortierella, Hebelomataceae*, and *Naucoria* (Fig. 4B).

**Figure 5.**
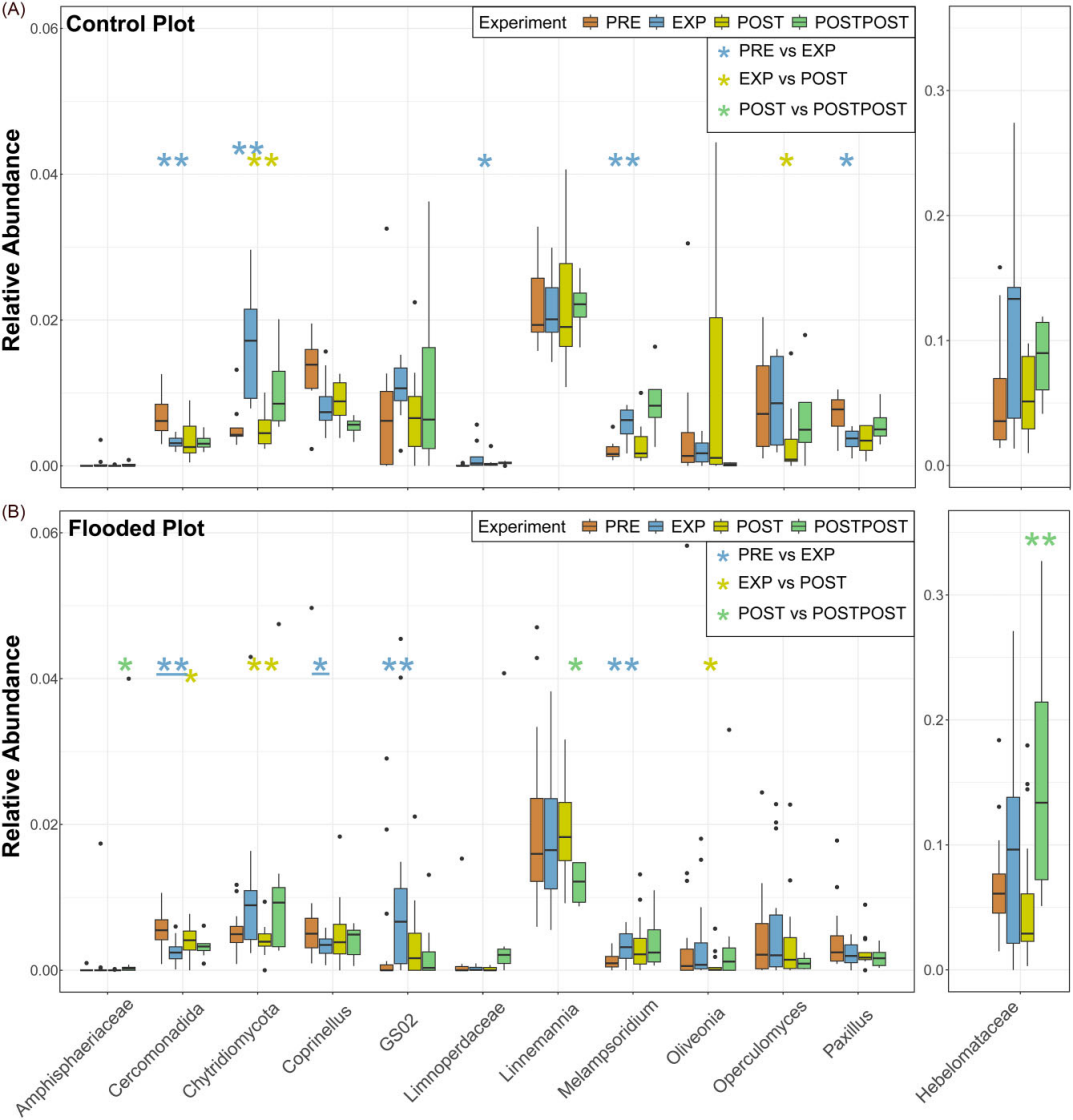
Significant changes in fungal genus relative abundances in the CP (A) and FP (B), based on sequencing data. The Wilcoxon rank-sum test determined statistically significant differences between periods (PRE vs. EXP, EXP vs. POST, and POST vs. POSTPOST). Asterisks (*) indicate statistical significance (*—*P* < .05, **—*P* < .01, and ***—*P* < .001), with blue, yellow, and green denoting changes between PRE-EXP, EXP-POST, and POST-POSTPOST, respectively. Line under the asterisks indicates Benjamini–Hochberg corrected (*P* < .05) Wilcoxon rank-sum test. Abbreviations: preflood period (PRE), flooding period (EXP), postflood period (POST), and a year after the flooding (POSTPOST).

During the flood in the FP, the relative abundance of *Rozellomycota* group *GS02* (formerly *Cryptomycota*; Tedersoo et al. 2017a, Kõljalg et al. 2020) and the relative abundance of *Melampsoridium* increased significantly (*P* < .01 and *P* < .05, respectively), while the relative abundance of *Coprinellus* decreased significantly (*P* < .05) (Fig. 5B). The relative abundance of *Cercomonadida* decreased significantly in both sampling areas during the flood (CP *P* < .01 and FP *P* < .01). In the CP, the relative abundances of *Chytridiomycota, Limnoperdaceae*, and *Melampsoridium* increased (*P* < .01, *P* < .05, and *P* <.01, respectively), while the relative abundance of *Paxillus* decreased during the flood (*P* < .05).

After the flood, the relative abundance of *Chytridiomycota* decreased significantly in both sampling areas (CP *P* <. 01 and FP *P* < .05). Similarly, in the FP, the relative abundance of *Oliveonia* decreased after the flood (*P* < .01). After the flood, the relative abundance of *Cercomonadida* increased, but the changes were significant only in the FP (*P* < .05). The relative abundance of *Operculomyces* decreased significantly after the flood in the CP (*P* < .05) but not in the FP. A year later, the relative abundances of *Hebelomataceae* and *Amphisphaeriaceae* increased significantly only in the FP (*P* < .01 and *P* < .05, respectively; Fig. 5B).

Opposite to CP, no significant changes were observed for *Limnoperdaceae, Paxillus*, and *Chytridiomycota* in FP. The most abundant fungal genera *Mortierella* relative abundance decreased during the flood and increased after the flood (Fig. 4B), but the changes were not statistically significant (Table S5). The proportions of *Mollisia* and *Peziza*, which each constituted <1% of the overall fungal community before the flood and in the CP, but the abundances increased during the flood in the FP and decreased again after the flood (Fig. 4B).

### Arbuscular mycorrhizal fungal relative abundance and diversity

**Figure 6.**
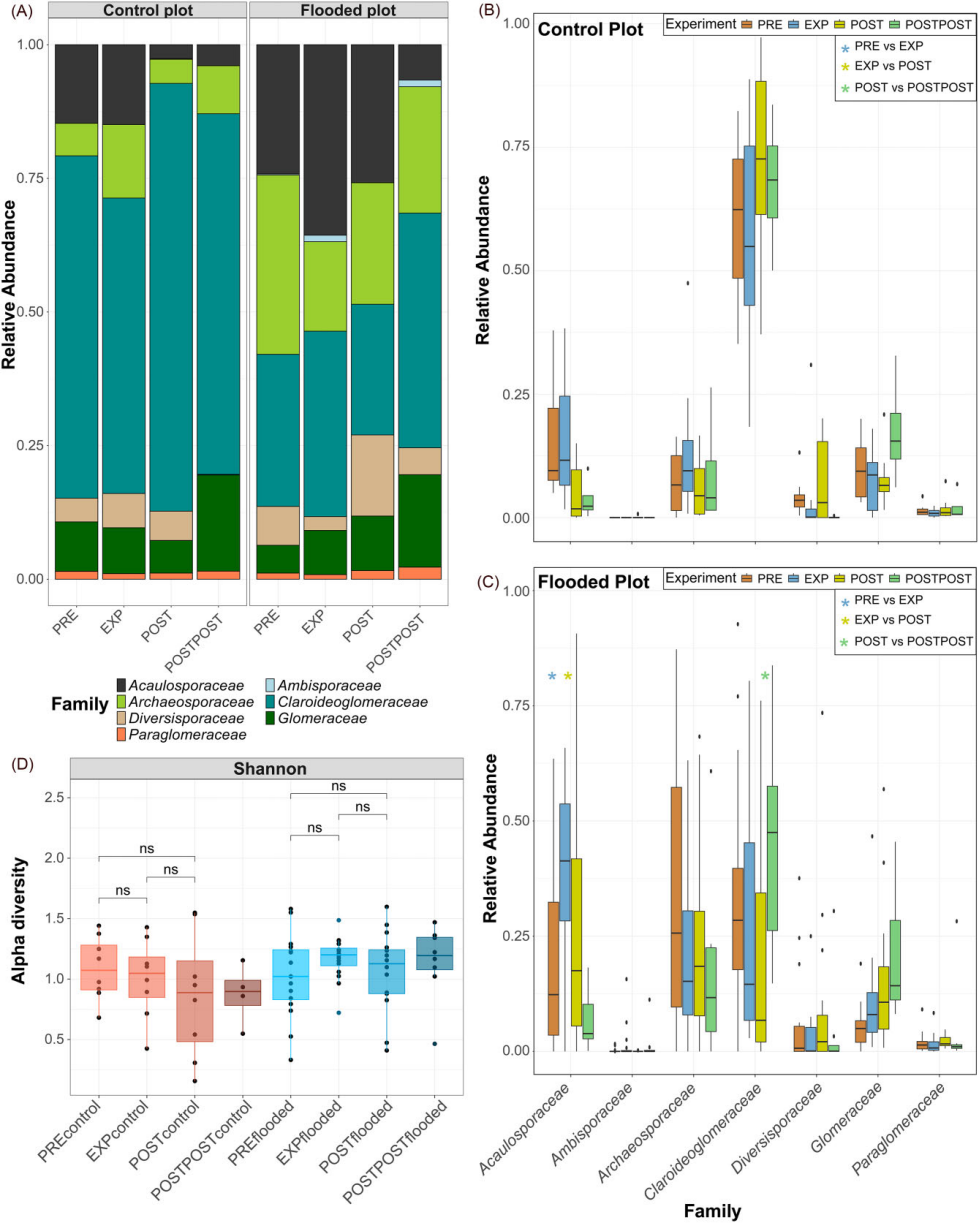
Relative abundances of AMF at the family (A) level in the CPs and FPs during different experiment periods, based on sequencing data. AMF families’ relative abundances in the CP (B) and FP (C) at different periods of the experiment. Panel D shows changes in the AMF Shannon diversity index during the experiment. Red bars represent CP, and blue bars represent FP. The Wilcoxon rank-sum test determined statistically significant differences between periods (PRE vs. EXP, EXP vs. POST, and POST vs. POSTPOST). Asterisks (*) indicate statistical significance (*—*P* < .05, **—*P* < .01, and ***—*P* < .001), with blue, yellow, and green denoting changes between PRE-EXP, EXP-POST, and POST-POSTPOST, respectively. Abbreviations: preflood period (PRE), flooding period (EXP), postflood period (POST), and a year after the flooding (POSTPOST).

In addition to all fungi, we specifically focused on AMF relative abundance, diversity, and their relation to environmental characteristics. Sequencing data revealed that AMF diversity did not change significantly during or after the flood (Fig. 6D), but PERMANOVA (Table S8) showed that treatment and experiment periods had a statistically significant effect (both *P* < .001) on the AMF community (Fig. S2). Treatment explained ~6.9% of the variance (*R*^2^ = 0.069), while experiment accounted for about 8.8% (*R*^2^ = 0.088). In agreement, NMDS ordination showed partial separation of FP and CP samples, rather than complete overlap (Fig. S10). Furthermore, the patterns in the ITS marker dataset for AMF were similar to those in the AMF marker dataset at the family level (Fig. S11).

At all time points, the AMF family with the highest relative abundance in the CP was *Claroideoglomeraceae* (Fig. 6A). In the FP, the dominant family shifted during the experiment, with *Archaeosporaceae* showing the highest relative abundance before the flood and *Acaulosporaceae* and *Claroideoglomeraceae* showing high relative abundances at other times (Fig. 6A). Experimental flooding increased the relative abundance of *Acaulosporaceae* in the FP, but there were no changes in CP (*P* < .05; Fig. 6B and C). After the flood, the relative abundance of the *Acaulosporaceae* decreased (*P* < .05) and *Claroideoglomeraceae* (not significantly) in FP. A year after the flooding (POSTPOST), the relative abundance of *Claroideoglomeraceae* in the FP increased (*P* < .05). There were no significant changes in the relative abundances of AMF families in CP during the experiment phases.

### Abundances of functional genes of the microbial nitrogen and methane cycles

**Figure 7.**
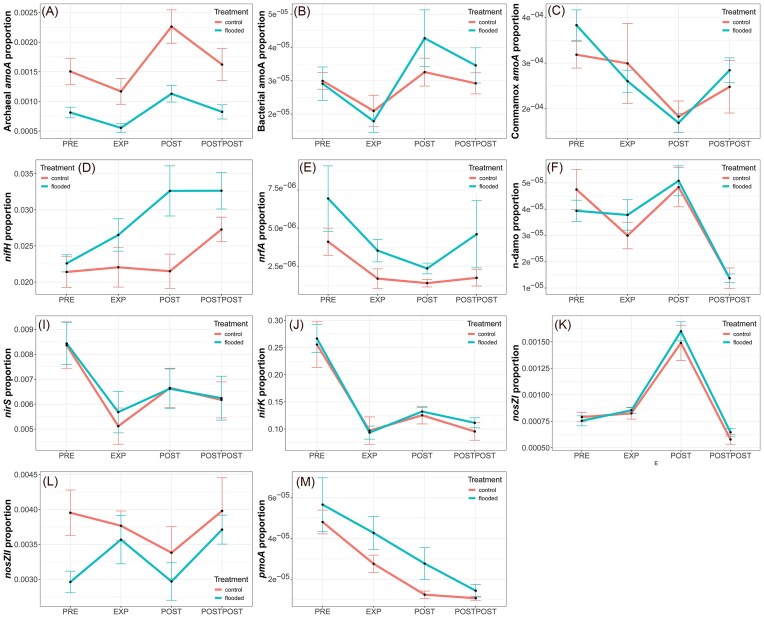
Changes of functional gene proportions in the CP and FP during different experiment periods, based on qPCR. Panels show archeal *amoA* (A), bacterial *amoA* (B), comammox *amoA* (C), *nifH* (D), *nrfA* (E), n-damo (F), *nirS* (I), *nirK* (J), *nosZI* (K), *nosZII* (L), and *pmoA* (M) functional gene proportions. Red lines show the changed of abundance proportion in the CP and blue in the FP. Black points on the line show the mean proportional abundance and errorbars the standard error of the mean. Abbreviations: preflood period (PRE), flooding period (EXP), postflood period (POST), and a year after the flooding (POSTPOST).

The significant changes in marker gene abundances between the sample areas are shown in Table S2, and changes between the experiment periods are shown in Tables S3 and S4. qPCR results showed, that archaeal *amoA* gene copy numbers were statistically lower in the FP than in CP (*P* < .05) throughout the experimental period (Fig. 7A). Flooding had a significant effect on archaeal *amoA* as the abundance decreased during the flood and increased after the flooding (*P* < .05). Bacterial *amoA* abundances significantly differed between the sampling plots before the flood (*P* < .05), but these differences disappeared after the flood (Fig. 7B). In the CP, changes in bacterial *amoA* abundance were not significant. In the FP, COMAMMOX *amoA* gene copy numbers decreased during and after the flood (Fig. 7C), and abundance before the flood differed significantly from the EXP and POST periods (*P* < .05). A year later (POSTPOST), the COMAMMOX *amoA* gene copy numbers had increased significantly in the FP (*P* < .05). The archaeal communities were characterized only by the abundance of archaeal 16S rRNA, archaeal *amoA*, and archaeal *mcrA* genes, and no archaeal community sequencing was performed.

Attempts to quantify the *mcrA* gene yielded no detectable amplification, indicating that the gene was either not present or occurred at levels below the detection limit. The abundance of *pmoA* gene copy numbers did not differ between the sample areas before the flooding (Fig. 7M). The flooding significantly affected *pmoA* abundance, with levels significantly higher in the FP than in the CP (*P* < .05).

The abundance of n-damo-specific 16S rRNA gene copy numbers was statistically lower a year after the flood (POSTPOST) (Fig. 7F). The POSTPOST period abundance of the n-damo-specific 16S rRNA genes was different from the PRE, EXP, and POST periods in the FP (*P* < .05). There were no statistically significant changes in n-damo-specific 16S rRNA gene abundances in the CP. *nifH* abundance changes in the FP were not significant, but *nifH* abundance increased with the flooding (Fig. 7D). The only significant changes in the FP were a year later, when the *nifH* abundance was statistically higher than the preflooding abundance and POST period (*P* < .05). The abundance of *nrfA* gene copies was significantly lower after the flood compared to preflood abundances in FP (*P* < .05) (Fig. 7E).

The abundance of *nirK* before the flood was statistically different from EXP, POST, and POSTPOST periods in FP (*P* < .05) (Fig. 7J). Similarly to the abundance of n-damo genes, flooding significantly affected fungal *nirK* gene copy numbers a year later. Fungal *nirK* abundance a year later was significantly higher than in the PRE, EXP, and POST periods at the FP (*P* < .05). There were no statistically significant changes in fungal *nirK* abundances in the CP. The abundance of nirS decreased significantly during the flood in FP (*P* < .05) and PRE period abundance was significantly lower from POST period (*P* < .05) (Fig. 7I). After the flood, the abundance of *nosZI* gene copies increased, and this change in abundance was significantly greater than PRE, EXP, or POSTPOST periods in the FP (*P* < .05) (Fig. 7K). The abundance of *nosZII* gene copies in the CP and the FP differed significantly before the flood (*P* < .05) (Fig. 7L). Statistically significant differences between the areas disappeared during the flood when *nosZII* abundance increased with the flood (not significantly). The abundance on *nosZII* decreased significantly after the flood (*P* < .05) and POST period abundance was significantly different (*P* < .05) form PRE on FP. The abundance of *nosZII* gene copy numbers increased significantly a year later at the FP (*P* < .05).

### Relationships between taxa, functional gene abundances, environmental characteristics, and GHG fluxes

**Figure 8.**
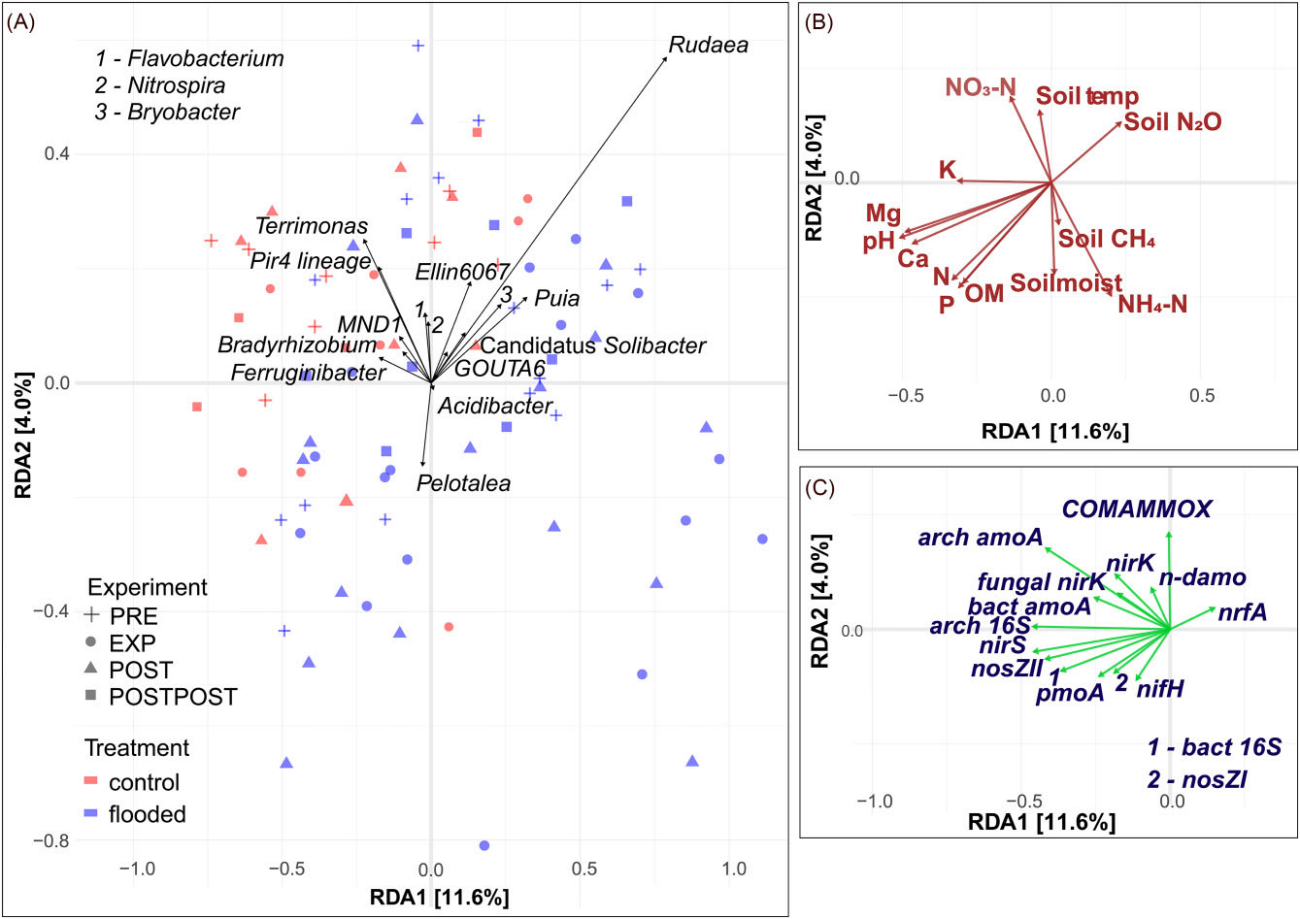
RDA ordination plots showing relationships of bacterial genera relative abundances (A) with soil physico-chemical properties (B), based on sequencing data and marker gene abundances (C), based on qPCR. The RDA was fitted for all variables together, and the biplots were separated into panels (A–C) later for readability. Abbreviations: nitrogen (N), phosphorus (P), soil organic matter (OM), ammonium (NH_4_-N), nitrate (NO_3_-N), soil methane emission (soil CH_4_), soil nitrous oxide emission (soil N_2_O), soil temperature (soil temp), potassium (K), magnesium (Mg), calcium (Ca), preflood period (PRE), flooding period (EXP), postflood period (POST), and a year after the flooding (POSTPOST).

Sequencing results suggested that bacterial genera *Terrimonas, Pir4 lineage, Nitrospira, Ellin6067*, and *MND1* were relatively more abundant in areas with higher soil NO_3_^−^ concentrations. From Fig. 8(B) and (C), the genera were positively related also with nitrification COMAMMOX *amoA, nirK* abundance, and soil temperature. On the other hand, *Acidibacter* was more abundant in areas with lower NO_3_^−^ concentrations and higher NH_4_^+^ concentrations (Fig. 8A and B). *Rudaea, Byrobacter, Puia, GOUTA6*, Candidatus *Solibacter, Ellin6067*, and *Nitrospira* in were more abundant in drier areas were positively related to soil N_2_O emissions (Fig. 8A and B). Abundances of the bacterial genera *Acidibacter* and *Pelotalea* were positively related to soil CH_4_ fluxes and soil moisture (Fig. 8A and B).

**Figure 9.**
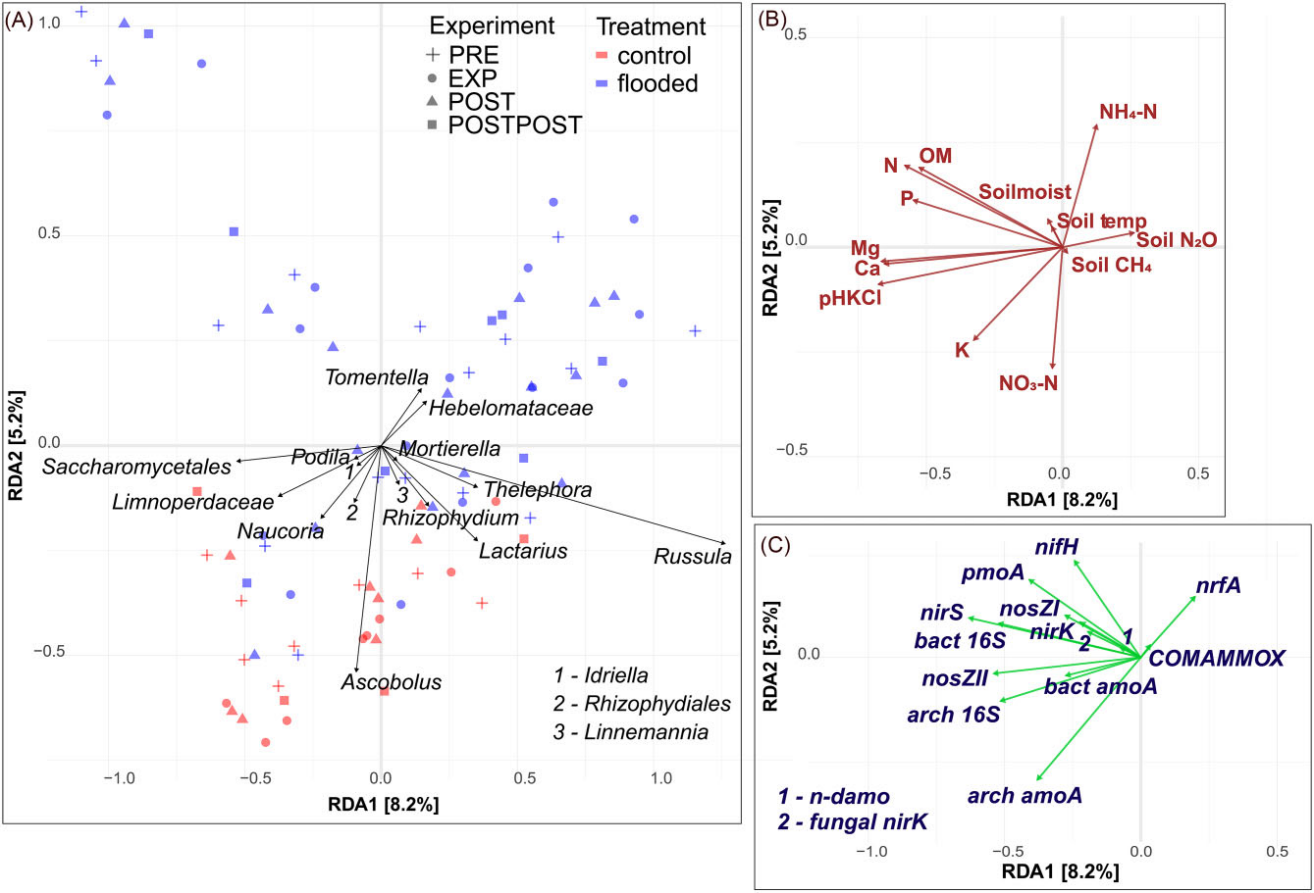
RDA ordination plots showing relationships of fungal genera relative abundances (A) with physico-chemical soil properties (B), based on sequencing data and marker gene abundances (C), based on qPCR. The RDA was fitted for all variables together, and the biplots were separated into panels (A–C) later for readability. Abbreviations: nitrogen (N), phosphorus (P), soil organic matter (OM), ammonium (NH_4_-N), nitrate (NO_3_-N), soil methane emission (soil CH_4_), soil nitrous oxide emission (soil N_2_O), soil temperature (soil temp), potassium (K), magnesium (Mg), calcium (Ca), preflood period (PRE), flooding period (EXP), postflood period (POST), and a year after the flooding (POSTPOST).

Fungal taxonomic groups *Tomentella* and *Hebelomataceae* were relatively more abundant in areas with higher soil NH_4_^+^ concentrations and positively related to soil N_2_O emission and soil moisture. *Tomentella* and *Hebelomatacea* were less abundant in areas with lower NO_3_^–^ and K concentrations. In contrast, *Ascobolus* and *Rhizophydiales* were more abundant in the opposite environment (Fig. 9A and B).

Fungal relative abundances of *Saccharomycetales, Podila, Idriella*, and *Limnoperdaceae* were positively correlated with soil Mg, Ca, and pH. Relative abundances of *Linnemannia, Rhizophydium*, and *Lactarius* more abundant in areas with soil CH_4_ emissions but RDA also showed that the genera were negatively associated with soil moisture (Fig. 9A and B). Among the analysed fungal genera, the genus *Russula* was most closely and positively associated with soil N_2_O emissions, and the fungal taxonomic group was also more abundant in soils with higher soil NH_4_^+^ concentrations (Fig. 9A and B).

**Figure 10.**
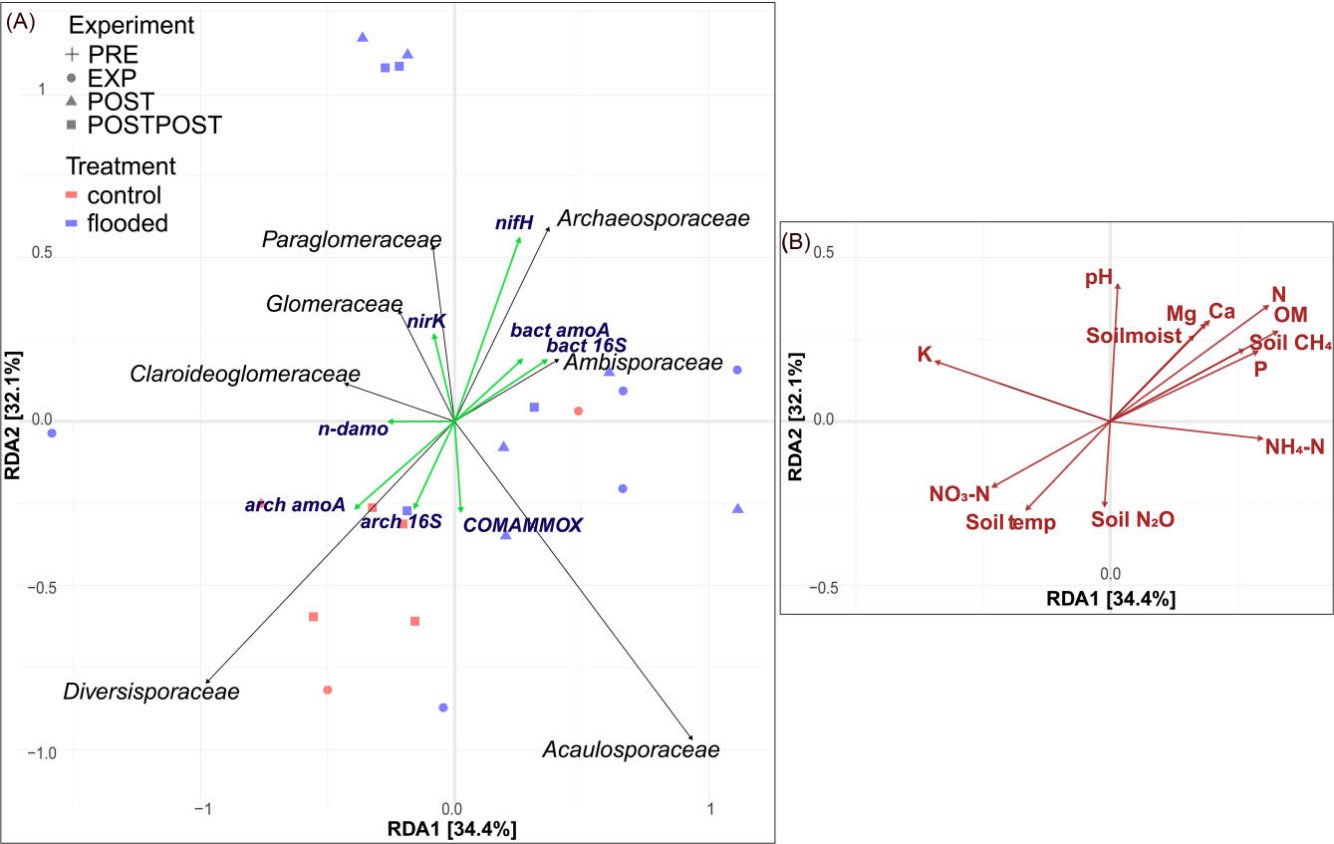
RDA ordination plots showing relationships of AMF families relative abundances (black lines), based on sequencing data with marker gene abundances (green lines), based on qPCR (A) and physico-chemical soil properties (B). The RDA was fitted for all variables together, and the biplots were separated into panels (A–C) later for readability. Abbreviations: nitrogen (N), phosphorus (P), soil organic matter (OM), ammonium (NH_4_-N), nitrate (NO_3_-N), soil methane emission (soil CH_4_), soil nitrous oxide emission (soil N_2_O), soil temperature (soil temp), potassium (K), magnesium (Mg), calcium (Ca), preflood period (PRE), flooding period (EXP), postflood period (POST), and a year after the flooding (POSTPOST).

AMF family *Diversisporaceae* was positively correlated with soil NO_3_^−^ concentration and temperature (Fig. 10A and B). *Archaeosporaceae* and *Ambisporaceae* were negatively related to soil NO_3_^−^ concentration and temperature, and positively related to Ca, Mg, N, OM, and P. Also, the relative abundance of *Archaeosporaceae* and *Ambisporaceae* were positively related to soil moisture and CH_4_ emissions*. Claroideoglomeraceae* were relatively more abundant in environments with higher soil K concentration. In contrast, *Paraglomeraceae* were more abundant in areas with higher pH. Among all the analysed AMF families, *Acaulosporaceae* and *Diversisporaceae* abundances were most closely associated with higher soil N_2_O emissions (Fig. 10A and B).

### Correlations between environmental characteristics, functional processes, microbial diversity, and GHG fluxes in the FPs

**Figure 11.**
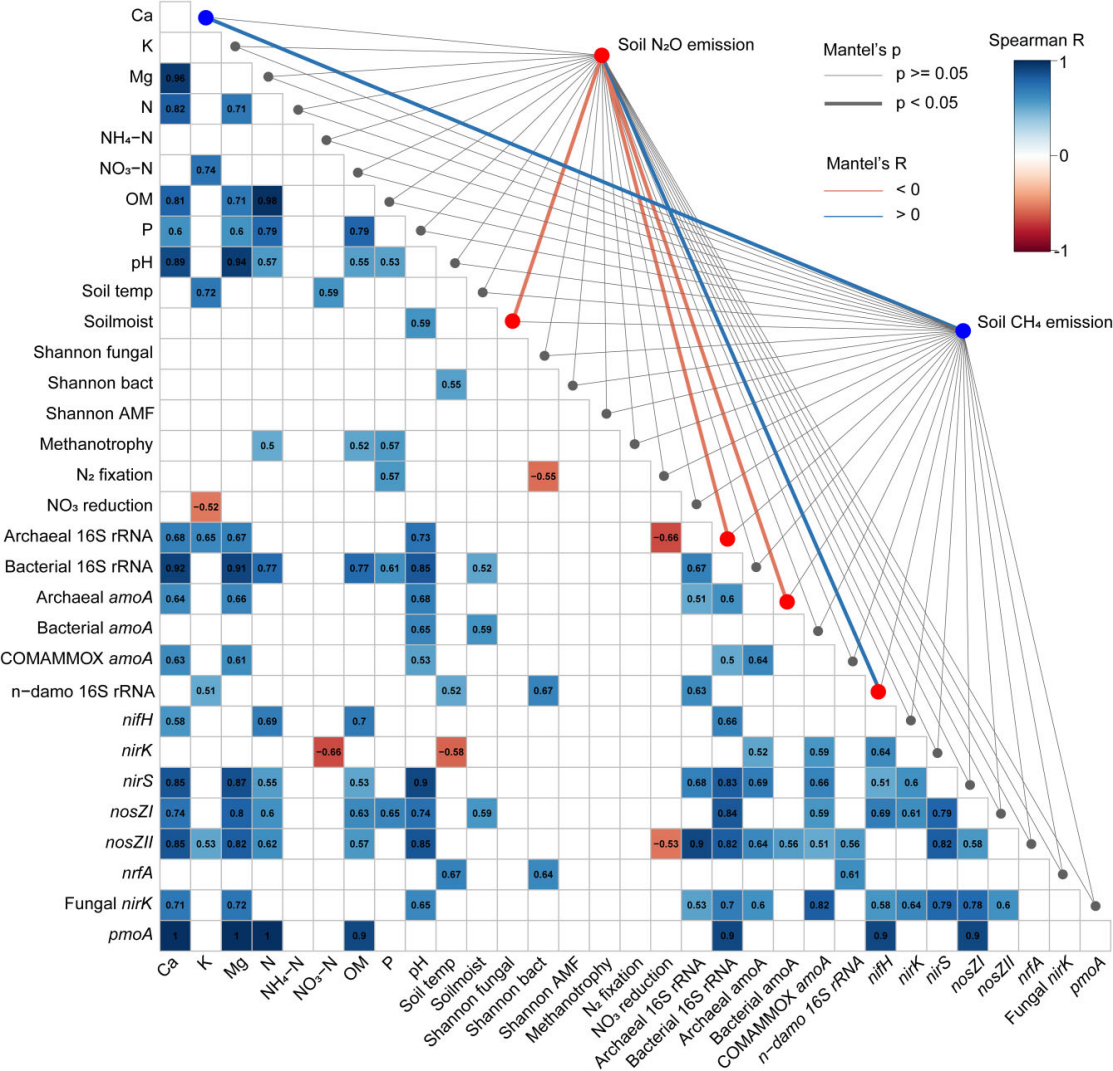
Spearman correlations and Mantel test during the FP flooding (EXP) period. The correlation table displays only significant (*P* < .05) correlations between environmental variables, marker genes, processes, and diversity. Mantel test results show soil N_2_O (red endpoints) and CH_4_ (blue endpoints) emissions in relation to environmental variables, functional genes (abundances determined with qPCR), processes (based on FAPROTAX), and diversity (based on sequencing data), represented by lines from *y*-axis. Thick lines indicate significant relationships (*P* < .05), with colour denoting positive (blue line) or negative (red line) relationships.

Correlation analysis showed that fungal diversity and soil K concentration were negatively correlated before the experimental flooding in FP (Fig. S4). During the flooding, bacterial diversity was positively correlated with temperature (Fig. 11). After the flooding, AMF diversity was positively correlated with soil NO_3_^–^ concentration (Fig. S5).

Soil moisture was positively correlated with the abundance of *pmoA* genes before the flooding (Fig. S4) and with bacterial *amoA* and *nosZI* abundances during the flooding in FP (Fig. 11). After the flooding, soil moisture was positively related to the abundance of bacterial *amoA, nosZII*, and bacterial 16S rRNA gene (Fig. S5). Additionally, soil moisture was negatively associated with soil NO_3_^–^ concentration but positively with NH_4_^+^ concentration after the flood in FP.

Mantel test results indicated that soil N_2_O emissions were negatively associated with bacterial 16S rRNA gene, bacterial *amoA, nirK, nirS*, and *nosZII* abundances before the flooding experiment (Fig. S4). During the flood, soil N_2_O emissions were negatively correlated with abundances of archaeal 16S rRNA and archaea *amoA* genes but positively with n-damo 16S rRNA gene abundance (Fig. 11). After the flooding, the negative correlations with archaeal abundance persisted, and N_2_O emissions were also negatively related to *nosZII* abundance (Fig. S5). Like RDA results, soil N_2_O emissions were negatively related to soil moisture during the flood (Fig. 10). Soil CH_4_ emissions were positively correlated with Ca during the flood (Fig. 11) and negatively correlated with *nifH* abundance after the flood (Fig. S5).

The processes showed that N_2_ fixation was negatively correlated with bacterial diversity during the flood but positively related with *nosZI* abundance after the flood. NO_3_^–^ reduction was negatively correlated with the archaeal 16S rRNA gene and *nosZII* abundance during the flood. Methanotrophy was positively correlated with soil N, Mg, and OM concentrations in the FP during the flood. After the flood, NO_3_^–^ reduction was negatively correlated with the abundance of *nirS, nosZII*, and fungal *nirK* genes but positively correlated with *nrfA* abundance (Fig. S5).

## Discussion

### Temporal changes in microbial diversity in response to flooding

Soil microbial communities in this study varied spatially and temporally in response to environmental characteristics. Floods and soil water content affect microbial communities and GHG emissions in riparian ecosystems (Schindler et al. 2020). Shen et al. (2021) observed lower soil bacterial α-diversity during flooding compared to drought. Similarly, we found that bacterial α-diversity was lower during the flood than before in both sample areas, with heavy rainfall at the end of the flooding experiment potentially influencing bacterial diversity in the CP. After the flooding event, the bacterial diversity recovered. Drainage and soil water content influence fungal community structure (Schimel et al. 1999, Graça et al. 2021), but fungi have been found to be more resistant to short-term flooding than bacteria (Wang et al. 2022a). Our results showed no significant fungal community changes during or after flooding. As in Unger et al. (2009), we assume that the flooding duration was insufficient to impact the fungal community significantly. Flood duration can be crucial for AMF diversity, with intensive flooding shown to have a negative impact and moderate flooding a positive one (Wang et al. 2011). AMF are aerobic microorganisms (Miller 2000) and AMF spores might not germinate under intensive or prolonged flooded conditions, which can slow down or stop the growth of AMF (Wang et al. 2011). We observed a slight but nonsignificant AMF diversity change under moderate flooding (Fig. 6D). In the case of vegetation, the flooding period may result in some herbaceous layer dieback and vigorous formation of adventitious roots in alder trees (Gill 1975, Ghanbary et al. 2012). The formation of adventitious roots is a classic response to soil anoxic conditions (McDonald et al. 2002).

### Spatial variation of microbial communities and nitrogen–carbon cycle genes

At our study site, soil moisture ranged between 0.4 and 0.7 m^3^/m^3^ (except POSTPOST period), while levels of 0.5–0.6 m^3^/m^3^ are the largest N_2_O source due to favourable conditions for N_2_O-forming processes, such as nitrification and denitrification (Bahram et al. 2022). Interpolation results showed that the soil was a source of N_2_O during the flood, particularly at drier points within the FP (points 3 and 5), which were slightly elevated. Topography influences N_2_O emissions by affecting soil hydrology (Fang et al. 2009, Vilain et al. 2010). At the water content levels of the drier FP points (0.4–0.5 m^3^/m^3^ water content), N_2_O production has been usually linked to nitrification (Congreves et al. 2019). Additionally, there was a negative correlation between N_2_O emissions and soil water content during the flood.

The relationship between soil water content and *pmoA* abundance may vary with environmental conditions, but similarly to Yang et al. (2024), we found no correlation between soil water content and *pmoA* abundance (determined by qPCR). Interpolation showed that the relative abundance of methanotrophic 16S rRNA taxa (FAPROTAX) was highest during the flood at points closest to the pumped water, which mimicked intensive rainfall and generated overland runoff. Before the flood, *pmoA* abundances did not differ significantly between CP and FP, but during the flood, abundance was significantly higher at the FP, indicating that pulsed flooding (water was pumped only during the day) affects *pmoA* abundance. Pulsed flooding supports CH_4_ oxidation in the topsoil (10 cm) more than continuous flooding (Chowdhury and Dick 2013). Additionally, methanotrophs can oxidize CH_4_ even under anaerobic conditions (Caldwell et al. 2008). Anaerobic CH_4_ oxidation can couple with different types of electron acceptors, like sulphate (Antler et al. 2014), iron (Oni and Friedrich 2017), nitrate, or nitrite (Zhang et al. 2021b).

We attempted to quantify the *mcrA* gene (qPCR), but no amplification was detected in the samples, indicating that the gene was either absent or present below the detection limit. Since methanogens are anaerobic, they are likely more abundant in deeper layers, or alternatively, the pulsed flooding or the duration of the flooding period may not have been long enough to create conditions favourable for the presence of the *mcrA* gene.

### Temporal shifts in the bacterial communities related to nitrogen and carbon cycling

**Figure 12.**
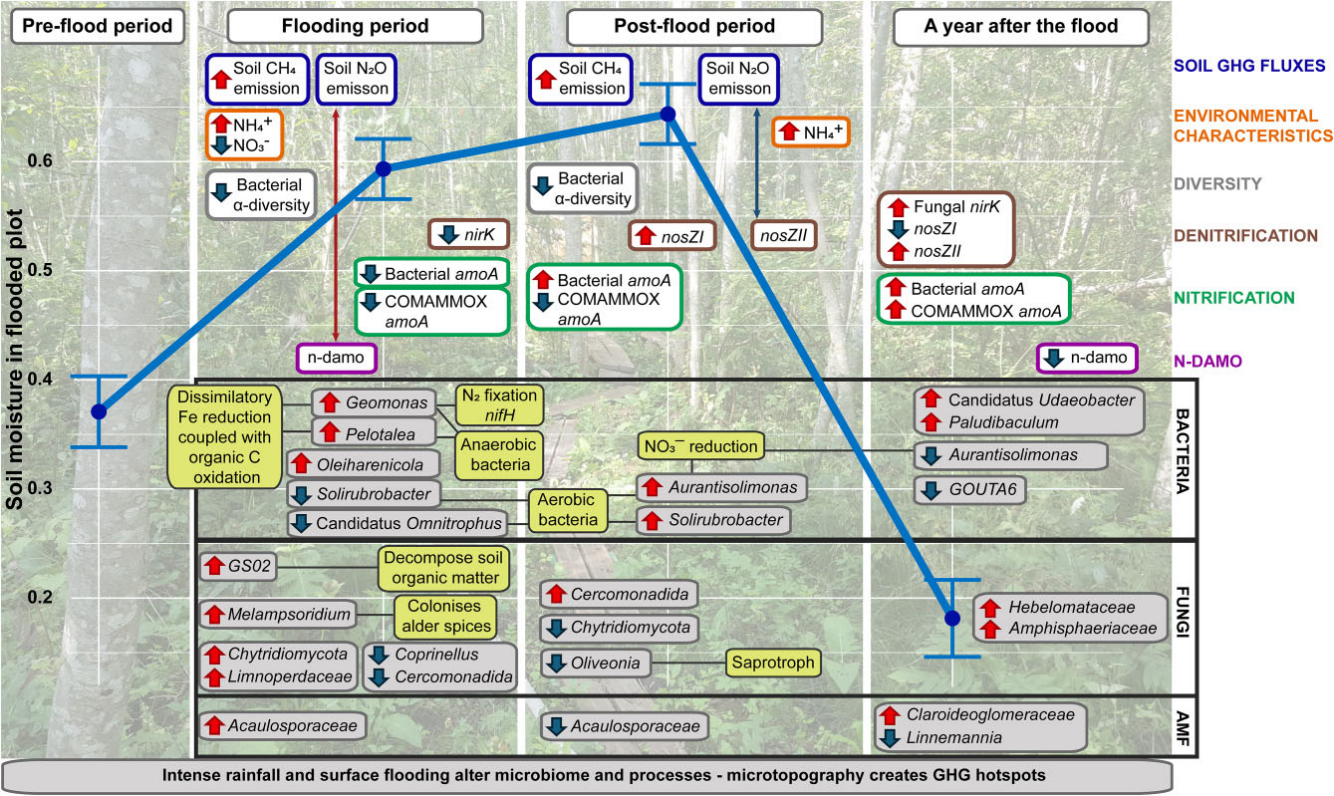
Main changes in the FP observed during the experiment (EXP), after the flood (POST), and 1-year postflood (POSTPOST). The blue line represents soil moisture. Red arrows indicate significant increases, while blue arrows indicate significant decreases (*P* < .05). Lines with two arrow ends show significant correlations (*P* < .05), red positive and blue negative correlation. Black lines highlight potential chemical or ecological traits of the genera that are shown in yellow boxes.

During the flooding period, soil water content increased significantly in the experimental plot (FP) (Schindler et al. 2020), leading to a shift in the bacterial community with a rise in anaerobic bacteria (Fig. 12). Sequencing data showed that flooding positively affected the strictly anaerobic genera *Pelotalea* and *Geomonas* (Itoh et al. 2021, Xu et al. 2021), which thrive in high-moisture habitats. These iron-reducing bacteria (Itoh et al. 2021, Xu et al. 2021) are linked to dissimilatory iron reduction coupled with organic C oxidation (Canfield et al. 1993, Zhao et al. 2024). Additionally, *Geomonas* is mainly found in paddy soils (Xu et al. 2019, Zhang et al. 2023), and some species can fix N (Xu et al. 2021).

Sequencing results showed that flooding significantly affected the aerobic microorganisms *Solirubrobacter* and *Aurantisolimonas* (Singleton et al. 2003, Liu et al. 2018). The relative abundance of *Solirubrobacter* decreased during the flooding, and it is typically found in extreme environments like deserts (Jiang et al. 2023) but also in agricultural soils (Singleton et al. 2003). The relative abundance of *Aurantisolimonas* increased after the flood. In this genus, a single species, *Aurantisolimonas haloimpatiens*, has been identified and is known for NO_3_^–^ reduction (Liu et al. 2018).

During the experiment, the relative abundance of *Bradyrhizobium* (Ormeño-Orrillo and Martínez-Romero 2019) increased significantly in CP but not in FP, which might suggest that the flood still affected *Bradyrhizobium* relative abundance. The N-fixing genus *Bradyrhizobium* is also linked to denitrification processes (Gao et al. 2021), with some species capable of complete denitrification and others associated with incomplete denitrification (Saeki et al. 2017).

The most abundant genus at our study site, *Flavobacterium*, is found in riparian areas (Chi et al. 2021). Some *Flavobacterium* species can oxidize carbohydrates (Bernardet and Bowman 2015), reduce NO_3_^–^ to NO_2_^–^ (Zhang et al. 2006), and one *Flavobacterium* species is capable of complete denitrification (Horn et al. 2005, Pishgar et al. 2019). It has been shown that *Flavobacterium* is an N_2_O-producing facultative aerobe (Pishgar et al. 2019), and sequencing results showed that with flooding, the abundance of *Flavobacterium* decreased, though not significantly. Also, *Flavobacterium* relative abundance was higher in habitats with greater NO_3_^–^ concentrations.

We analysed the N-mediated n-damo process, an N-cycle process linked to the C cycle, involving the anaerobic bacterium Candidatus *Methylomirabilis oxyfera*. Candidatus *Methylomirabilis oxyfera* reduces NO_2_^–^ to N_2_ without N_2_O reductase, producing oxygen for CH_4_ oxidation (Ettwig et al. 2010). N-damo-specific 16S rRNA gene abundance (determined by qPCR) was positively correlated with N_2_O emissions in the FP during the flood. However, our results showed that n-damo-specific 16S rRNA gene abundance did not change significantly during or after the flood, and the genus Candidatus *Methylomirabilis* was not found in the sample area.

### Alteration of nitrogen-cycle gene abundances and associated N_2_O and N_2_ fluxes

Our results suggest a slowdown of nitrification in some locations during the flood. N_2_O emissions are typically linked to soil NO_3_^–^ availability (Vilain et al. 2010, Pärn et al. 2018), which accumulates in the soil through nitrification. Our findings show a decrease in soil NO_3_ concentration and a decrease in the relative abundance of nitrification-associated bacteria *Nitrospira* (sequencing data), which belongs to the *Nitrosomonadaceae* family and is NH_3_ oxidizer (Prosser et al. 2014, Yan et al. 2023) and prefers lower soil water content. Additionally, qPCR results revealed that bacterial and COMAMMOX *amoA* abundance decreased during flooding. *Nitrospira*, known for the comammox process (Wang et al. 2019), decreased in both plots, but the decrease was greater in FP. However, sequencing data showed that flooding increased the relative abundance of AOB from *MND1* and *Ellin6067* genera (not significantly). *Ellin6067* is a main nitrifier in aquaculture ponds, and AOB-driven nitrification has been shown to be the primary source of N_2_O in those environments (Deng et al. 2024).

Denitrification is an anaerobic four-step process in which NO_3_^–^ is reduced to N_2_ (Kleineidam et al. 2025)_._ The *nirK* and *nirS* genes serve as markers and show potential metabolic capacity for the NO_2_^–^ reduction process (Kuypers et al. 2018). We observed a negative correlation between *nirK* and soil NO_3_^–^ concentration during the flood. Microorganisms that complete the final denitrification step, reducing N_2_O to N_2_, use N_2_O reductase *nosZ* clades I and II (Hallin et al. 2018). Microorganisms possessing *nosZI* genes are more likely to be complete denitrifiers, as they are typically found alongside *nirK* or *nirS* (Graf et al. 2014). Also, nearshore areas have a low N_2_O-producing capability due to the dominance of *nosZI*-harbouring bacteria, which are N_2_O-reducing microorganisms (Zhao et al. 2022). Interpolation showed that points with higher soil water content acted as weak N_2_O sinks. Flooding increased the abundance of *nosZI* and *nosZII*, with *nosZI* positively correlated with soil water content. Additionally, the average soil water content in the FP (except at points 3 and 5) was above 0.6 m^3^/m^3^, suggesting conditions favourable for complete denitrification (Vilain et al. 2010).

DNRA is an anaerobic process in OM-rich soils (Rütting et al. 2011), but it can be limited by NO_3_^–^ availability (Silver et al. 2001). FP’s interpolation, based on qPCR results, showed higher *nrfA* gene abundance than CP during the flood (Fig. S7E). After the flood, the abundance of *nrfA* was positively correlated with NO_3_^–^ reduction (Fig. S5), suggesting DNRA process activity after the flooding experiment.

Flooding positively influenced microorganisms harbouring the *nifH* gene, indicating a potential metabolic capacity for nitrogen fixation. *nifH* abundance (qPCR data) correlated positively with soil water content, and interpolation, based on qPCR results, showed the highest *nifH* gene abundances at the points closest to the water source (Fig. S6F). Sequencing revealed that flooding significantly increased the relative abundance of *Geomonas* (Fig. 11), potentially N_2_-fixer (Ormeño-Orrillo and Martínez-Romero 2019, Liu et al. 2023) carrying the *nifH* gene (Liu et al. 2023).

### Temporal impacts of flooding on fungal community structure

Similarly to previous studies, we did not see a significant change in the fungal community, but the relative abundance of *Rozellomycota* group GS02, *Coprinellus, Melampsoridium, Chytridiomycota*, and *Oliveonia, Cercomonadida* changed significantly during or after the flood in the FP (Fig. 11).

Flooding significantly increased the relative abundance of GS02 and *Melampsoridium* fungi. Clade GS02, an ectomycorrhizal fungus (EMF) in the *Rozellomycota* (*Cryptomycota*) phylum, has been found in pine stands (Dewi et al. 2016) and near-neutral temperate soils in Europe (Tedersoo et al. 2017b). The *Rozellomycota* group GS02 has been shown to have cellulase activities (Bhosale et al. 2024) important in decomposing soil OM (Daunoras et al. 2024).

Flooding significantly increased the relative abundance of *Melampsoridium* (Fig. 11). *Melampsoridium* is a rust fungus (*M. hiratsukanum*) that colonizes alder species (Hantula et al. 2009) and can impair leaf physiology and photosynthesis, reducing their ability to fix C (Carretero et al. 2011, Gortari et al. 2018). On the other hand, flooding significantly decreased the relative abundance of *Coprinellus and Cercomonadida* genera. *Coprinellus* has been identified under aerobic conditions and has been shown to be affected by floods (Tian et al. 2015).

The relative abundance of soil saprotroph genera *Oliveonia* (Trofymow et al. 2020) and *Chytridiomycota* decreased significantly after the flood. *Chytridiomycota* are primarily aquatic fungi that can grow on organic material in soils (Barr 2001), and some are able to use NO_3_^–^ as a sole source of nitrogen (Digby et al. 2010).

The relative abundance of *Cortinarius* increased with flooding as soil NO_3_^–^ levels declined. This nitrophobic genus (Lilleskov et al. 2002, Bashian-Victoroff et al. 2025) is found in flooded areas (Sumorok et al. 2008) and wetlands with low soil N availability (Filippova and Thormann 2014, Aučina et al. 2019). The flood increased slightly the relative abundance of genera *Naucoria* and *Russula*, both mycorrhizal associates of alder roots (Moreau 2005). It also increased *Tomentella* abundance, a common genus in the alder microbiome (Schwob et al. 2017, Fuller et al. 2023). Alders form symbiotic relationships with EMF and AMF, which aid nutrient and water uptake and enhance resilience to environmental stress (Fuller et al. 2023).

The most abundant AMF families at the study site were *Claroideoglomeraceae* and *Archaeosporaceae*. During the flood, the relative abundance of the family *Acaulosporaceae* increased significantly (Fig. 11). This family occurs in several different habitats, like tropical forests (Leal et al. 2013), is stress-tolerant (Chagnon et al. 2013) and prefers acidic soils with low soil bulk density and higher N content (Veresoglou et al. 2013, Davison et al. 2021). Flooding and high soil water content can increase bulk density, and fine-textured soils, like the clay soils in this study, are prone to deformation and compaction (Saffih-Hdadi et al. 2009).

## Conclusions

Microbiome studies are essential for understanding how soil communities respond and adapt to climate change, particularly in regions where flooding is expected to increase. The average temperature during the experiment ranged from 19.9 ± 2.1°C, and the flood plot was significantly higher in soil moisture. Mid-growing-season flooding significantly impacted soil microbial communities (Fig. 11), particularly the C and N cycles and GHG fluxes. The first hypothesis was partially supported, as bacterial diversity declined during the flood, whereas fungal diversity did not significantly change during or after the flood, confirming its resistance to short-term environmental changes. Among AMF, only *Acaulosporaceae* abundance increased significantly, reinforcing also AMF’s resilience to flooding. The second hypothesis proposed that changes in the microbiome are responsible for decreases in N_2_O emissions and increases in CH_4_ emissions after flooding. We saw that soil water content fluctuations led to CH_4_ consumption and emission in FP, favouring n-damo bacteria. The n-damo gene was also positively correlated with soil N_2_O emission; however, the genus Candidatus *Methylomirabilis*, which could carry out anaerobic methane oxidation with nitrite reduction, was not detected. The *pmoA* gene, linked to methanotrophic activity, was more abundant in FP, aligning with areas of high CH_4_ oxidation. Also, *mcrA* gene was not detected in the samples. Methanogens are anaerobic and most likelely more abundant in deeper layers, or the flooding period may not have been long enough to create favourable conditions. Lower soil water content at N_2_O emission spots suggested nitrification as the primary N_2_O source. Second hypothesis was therefore partially correct as we saw that flooding created N_2_O and CH_4_ emission hot spots, but the emissions were influenced by soil water content and microtopography. The third hypothesis was also partially true, as soil water content was positively correlated with bacterial *amoA*, while the relative abundances of nitrifiers *MND1* and *Ellin6067* increased. On the other hand, the comammox process decreased with flooding as the relative abundance of *Nitrospira* and COMAMMOX *amoA* gene copies decreased. Flooding positively influenced alder-associated fungi (*Naucoria, Russula*, and *Tomentella*) and N_2_-fixing bacteria (*Bradyrhizobium* and *Geomonas*), with *nifH* gene abundance highest at points closest to the water source.

The pulsing nature and duration of short-term flooding and microtopography influenced soil microbial communities and processes, underscoring the intricate interplay between hydrology, soil microbiology, and GHG fluxes. This study highlights the need for further experiments to examine microgradients between alder roots and bulk soil.

## Supplementary Material

fiaf109_Supplemental_File

## Data Availability

The data underlying this article are available in European Nucleotide Archive at https://www.ebi.ac.uk/ena/browser/home, and can be accessed with the primary accession code PRJEB86668.
