# Supplementary material for: Temporal and spatial dynamics of microbial communities and greenhouse gas flux responses to experimental flooding in riparian forest soils
Source: FEMS Microbiol Ecol. 2025 Oct 25;101(12):fiaf109. doi: 10.1093/femsec/fiaf109 (PMC12603561; doi:10.1093/femsec/fiaf109)
Supplement: fiaf109_Supplemental_File [file fiaf109_supplemental_file.docx]

**Temporal and spatial dynamics of microbial communities and greenhouse gas flux responses to experimental flooding in riparian forest soils**

Kristel Reiss^1^, Ülo Mander^1^, Maarja Öpik^2^, Siim Kaarel Sepp^2,3^, Kärt Kanger^1^, Thomas Schindler^1^, Kaido Soosaar^1^, Mari Pihlatie^4,5^, Klaus Butterbach-Bahl^6^, Anuliina Putkinen^4,5^, Ülo Niinemets^7^, Mikk Espenberg^1^

^1^Institute of Ecology and Earth Sciences, University of Tartu, Vanemuise 46, Tartu 51003, Estonia

^2^Institute of Ecology and Earth Sciences, University of Tartu, J. Liivi 2, Tartu 51003, Estonia

^3^Netherlands Institute of Ecology (NIOO-KNAW), Droevendaalsesteeg 10, 6708 PB Wageningen, The Netherlands

^4^Department of Agricultural Sciences, University of Helsinki, PO Box 56, Helsinki 00014, Finland

^5^Institute for Atmospheric and Earth System Research, Faculty of Agriculture and Forestry, University of Helsinki, PO Box 56, Helsinki 00014, Finland

^6^Department of Agroecology, Land-CRAFT, Center for Landscape Research in Sustainable Agricultural Futures, Aarhus University, Blichers Allé 20, 8830 Tjele, Denmark

^7^Chair of Crop Science and Plant Biology, Estonian University of Life Sciences, Kreutzwaldi 1, 51006 Tartu, Estonia

*Correspondence to*: Kristel Reiss (kristel.reiss@ut.ee)

**Supplementary Table 1.** The primers for functional genes, primer concentrations, qPCR programs used, and used ranges of standards.

| **Functional gene** | **Primer** | **Primer reference** | **Amplicon size (bp)** | **Primer concentration (µM)** | **qPCR program** | **Range of standard; amplification efficiency** |
| --- | --- | --- | --- | --- | --- | --- |
| Bacterial 16 rRNA | Bact517F | Liu et al., 2007 | 530 | 0.6 | 95°C 10 min; 35 cycles: 95°C 30 s; 60°C 45 s; 72°C 45s | 10^6^; 1.837 ± 0.04 |
|  | Bact1028R | Dethlefsen et al., 2008 |  |  |  |  |
| Archaeal 16S rRNA | Arc519F | Espenberg et al., 2016 | 393 | 0.6 | 95°C 10 min; 45 cycles: 95°C 15 s; 56°C 30 s; 72°C 30s | 10^4^ – 10^5^; 1,861 ± 0.081 |
|  | Arch910R |  |  |  |  |  |
| *nirS* | nirSCd3af | Kanter et al., 2016 | 387 | 0.8 | 95°C 10 min; 45 cycles: 95°C 15 s; 55°C 30 s; 72°C 30s, 80°C 30 s | 10^3^ – 10^4^; 1.72 ± 0.053 |
|  | nirSR3cd |  |  |  |  |  |
| *nirK* | nirK876 | Hallin & Lindgren, 1999 | 165 | 0.8 | 95°C 10 min; 45cycles: 95°C 15 s; 58°C 30 s; 72°C 30s, 80°C 30 s | 10^4^ – 10^6^; 1.687 ± 0.065 |
|  | nirK1040 |  |  |  |  |  |
| *nosZI* | nosZ2F | Henry et al., 2006 | 267 | 0.6 | 95°C 10 min; 45cycles: 95°C 15 s, 60°C 30 s, 72°C 30 s, 80°C 30 s | 10^2^ – 10^3^; 1.171 ± 0.079 |
|  | nosZ2R |  |  |  |  |  |
| *nosZII* | nosZ-II-F | Jones et al., 2013 | ~700 | 0.6 | 95°C 10 min; 45 cycles: 95°C 30 s, 54°C 45 s, 72°C 45 s, 80°C 45 s | 10^7^ – 10^8^; 1.618 ± 0.07 |
|  | nosZ-II-R |  |  |  |  |  |
| *nifH* | Ueda19F | Ueda et al., 1995 | 390 | 0.8 | 95°C 10 min; 45 cycles: 95°C 30 s, 53°C 45 s, 72°C 45 s | 10^4^ – 10^5^; 1.795 ± 0.038 |
|  | Ueda407R |  |  |  |  |  |
| *nrfA* | 6RF | Takeuchi, 2006 | 222 | 0.8 | 95°C 10 min; 45 cycles: 95°C 15 s, 55°C 30 s, 72°C 30 s | 10^0^ – 10^2^; 1.77 ± 0.122 |
|  | 6R |  |  |  |  |  |
| Bacterial *amoA* | amoA-1F | Rotthauwe et al., 1997 | 491 | 0.8 | 95°C 10 min; 45 cycles: 95°C 30 s, 57°C 45 s, 72°C 45 s | 10^0^ – 10^2^; 1.855 ± 0.038 |
|  | amoA-2R |  |  |  |  |  |
| Archaeal *amoA* | CrenamoA 23F | Tourna et al., 2008 | ~600 | 0.8 | 95°C 10 min; 45 cycles: 95°C 30 s, 55°C 45 s, 72°C 45 s | 10^0^ – 10^2^; 1.836 ± 0.046 |
|  | CrenamoA 616R |  |  |  |  |  |
| COMAMMOX *amoA* | comamoA AF | Wang et al., 2018 | 436 | 0.8 | 95°C 10 min; 40 cycles: 95°C 15 s, 55°C 30 s, 72°C 30 s | 10^2^ – 10^3^; 1.841 ± 0.046 |
|  | comamoA SR |  |  |  |  |  |
| Fungal *nirK* | FnirK-F3 | Chen et al., 2016 | 233 | 0.8 | 95°C 10 min; 45 cycles: 95°C 15 s, 56°C 30 s, 72°C 30 s | 10^3^ – 10^6^; 1.731 ± 0.048 |
|  | FnirK-R2 |  |  |  |  |  |
| N-damo 16 rRNA | pq2F | Ettwig et al., 2009 | 281 | 0.8 | 95 °C 10 min, 45 cycles: 95 °C 15 s, 60°C 30 s, 72 °C 30 s, 80 °C 30 s | 10^2^ – 10^3^, 1.779 ± 0.054 |
|  | pq2R |  |  |  |  |  |
| *mcrA* | mcrA-F | Espenberg et al., 2016 | 135 | 0.8 | 95 °C 10 min, 55 cycles: 95 °C 15 s, 51°C 30 s, 72 °C 30 s | Not detected |
|  | mcrA-R |  |  |  |  |  |
| *pmoA* | A189F | Costello and Lidstrom, 1999 | 510 | 0.6 | 95 °C 10 min, 35 cycles: 95 °C 15 s, 57 °C 30 s, 72 °C 30 s | 50 – 10^7^; 1.860 ± 0.049 |
|  | Mb661R |  |  |  |  |  |

**Supplementary Table 2:** Significant differences in marker gene abundances between the control plot (CP) and the flooded plot (FP). The calculation of marker genes has been logarithmised, followed by applying the Welch Two Sample t-test. Significant differences (p<0.05) are shown in red. Abbreviations: pre-flood period (PRE), flooding (EXP), post-flood period (POST), a year after the flooding (POSTPOST).

| **Gene** | **Experiment** | **Mean CP** | **Mean FP** | **p-value** |
| --- | --- | --- | --- | --- |
| Archaeal 16S rRNA | PRE | 29.36 | 28.92 | >0.05 |
| Archaeal 16S rRNA | EXP | 28.85 | 28.47 | >0.05 |
| Archaeal 16S rRNA | POST | 28.89 | 28.40 | <0.05 |
| Archaeal 16S rRNA | POSTPOST | 29.56 | 28.94 | >0.05 |
| Bacterial 16S rRNA | PRE | 33.98 | 33.80 | >0.05 |
| Bacterial 16S rRNA | EXP | 33.95 | 33.83 | >0.05 |
| Bacterial 16S rRNA | POST | 33.59 | 33.48 | >0.05 |
| Bacterial 16S rRNA | POSTPOST | 34.29 | 34.09 | >0.05 |
| Archaeal *amoA* | PRE | 24.55 | 23.46 | <0.01 |
| Archaeal *amoA* | EXP | 24.02 | 22.72 | <0.05 |
| Archaeal *amoA* | POST | 24.74 | 23.56 | <0.01 |
| Archaeal *amoA* | POSTPOST | 25.02 | 23.76 | <0.05 |
| Bacterial *amoA* | PRE | 18.97 | 18.58 | <0.05 |
| Bacterial amoA | EXP | 18.09 | 17.78 | >0.05 |
| Bacterial amoA | POST | 18.66 | 18.74 | >0.05 |
| Bacterial amoA | POSTPOST | 19.26 | 19.15 | >0.05 |
| COMAMMOX *amoA* | PRE | 22.36 | 22.40 | >0.05 |
| COMAMMOX *amoA* | EXP | 21.84 | 21.87 | >0.05 |
| COMAMMOX *amoA* | POST | 21.05 | 20.85 | >0.05 |
| COMAMMOX *amoA* | POSTPOST | 22.22 | 22.30 | >0.05 |
| n-damo 16S rRNA | PRE | 19.54 | 19.09 | >0.05 |
| n-damo 16S rRNA | EXP | 18.80 | 18.95 | >0.05 |
| n-damo 16S rRNA | POST | 19.10 | 19.10 | >0.05 |
| n-damo 16S rRNA | POSTPOST | 18.00 | 17.90 | >0.05 |
| *nifH* | PRE | 28.44 | 28.35 | >0.05 |
| *nifH* | EXP | 28.38 | 28.57 | >0.05 |
| *nifH* | POST | 28.03 | 28.48 | >0.05 |
| *nifH* | POSTPOST | 29.14 | 29.16 | >0.05 |
| *nirK* | PRE | 31.93 | 31.84 | >0.05 |
| *nirK* | EXP | 30.18 | 30.25 | >0.05 |
| *nirK* | POST | 30.54 | 30.57 | >0.05 |
| **Gene** | **Experiment** | **Mean CP** | **Mean FP** | **p-value** |
| *nirK* | POSTPOST | 30.89 | 30.92 | >0.05 |
| *nirS* | PRE | 27.07 | 26.85 | >0.05 |
| *nirS* | EXP | 26.26 | 26.18 | >0.05 |
| *nirS* | POST | 26.34 | 26.14 | >0.05 |
| *nirS* | POSTPOST | 26.98 | 26.71 | >0.05 |
| *nosZI* | PRE | 23.71 | 23.43 | >0.05 |
| *nosZI* | EXP | 23.73 | 23.66 | >0.05 |
| *nosZI* | POST | 24.20 | 24.20 | >0.05 |
| *nosZI* | POSTPOST | 23.58 | 23.51 | >0.05 |
| *nosZII* | PRE | 26.02 | 25.42 | <0.05 |
| *nosZII* | EXP | 25.93 | 25.64 | >0.05 |
| *nosZII* | POST | 25.38 | 25.04 | >0.05 |
| *nosZII* | POSTPOST | 26.34 | 26.04 | >0.05 |
| *nrfA* | PRE | 15.94 | 16.18 | >0.05 |
| *nrfA* | EXP | 14.26 | 15.17 | >0.05 |
| *nrfA* | POST | 13.98 | 14.58 | >0.05 |
| *nrfA* | POSTPOST | 15.03 | 15.61 | >0.05 |
| Fungal *nirK* | PRE | 21.37 | 21.06 | >0.05 |
| Fungal *nirK* | EXP | 21.06 | 20.65 | >0.05 |
| Fungal *nirK* | POST | 21.10 | 20.90 | >0.05 |
| Fungal *nirK* | POSTPOST | 21.52 | 22.15 | >0.05 |
| *pmoA* | PRE | 19.79 | 21.05 | >0.05 |
| *pmoA* | EXP | 18.91 | 20.60 | <0.05 |

**Supplementary Table 3.** Friedman post-hoc pairwise Wilcoxon test between different study period marker gene abundances (qPCR) in flooded plot. Only statistically significant results are shown in the table. Adjusted p-values below 0.05 were considered statistically significant (p.adj.signif). Abbreviations: pre-flood period (PRE), flooding (EXP), post-flood period (POST), a year after the flooding (POSTPOST).

|  | Treatment | group1 | group2 | n1 | n2 | statistic | p | p.adj | p.adj.signif |
| --- | --- | --- | --- | --- | --- | --- | --- | --- | --- |
| **archeal 16S** | flooded | POST | POSTPOST | 8 | 8 | 0 | 0.008 | 0.023 | * |
|  | flooded | POST | PRE | 8 | 8 | 0 | 0.008 | 0.023 | * |
| **bacterial 16S** | flooded | EXP | POST | 8 | 8 | 35 | 0.016 | 0.019 | * |
|  | flooded | EXP | POSTPOST | 8 | 8 | 1 | 0.016 | 0.019 | * |
|  | flooded | POST | POSTPOST | 8 | 8 | 0 | 0.008 | 0.016 | * |
|  | flooded | POST | PRE | 8 | 8 | 0 | 0.008 | 0.016 | * |
|  | flooded | POSTPOST | PRE | 8 | 8 | 36 | 0.008 | 0.016 | * |
| **archaeal *amoA*** | flooded | EXP | POST | 8 | 8 | 0 | 0.008 | 0.023 | * |
|  | flooded | EXP | POSTPOST | 8 | 8 | 0 | 0.008 | 0.023 | * |
|  | flooded | EXP | PRE | 8 | 8 | 2 | 0.023 | 0.047 | * |
| **bacterial *amoA*** | flooded | EXP | POSTPOST | 8 | 8 | 0 | 0.008 | 0.047 | * |
| **COMAMMOX *amoA*** | flooded | EXP | POST | 8 | 8 | 36 | 0.008 | 0.016 | * |
|  | flooded | EXP | POSTPOST | 8 | 8 | 2 | 0.023 | 0.028 | * |
|  | flooded | EXP | PRE | 8 | 8 | 1 | 0.016 | 0.023 | * |
|  | flooded | POST | POSTPOST | 8 | 8 | 0 | 0.008 | 0.016 | * |
|  | flooded | POST | PRE | 8 | 8 | 0 | 0.008 | 0.016 | * |
| **n-damo** | flooded | EXP | POSTPOST | 8 | 8 | 36 | 0.008 | 0.016 | * |
|  | flooded | POST | POSTPOST | 8 | 8 | 36 | 0.008 | 0.016 | * |
|  | flooded | POSTPOST | PRE | 8 | 8 | 0 | 0.008 | 0.016 | * |
| ***nifH*** | flooded | POST | POSTPOST | 8 | 8 | 0 | 0.008 | 0.047 | * |
|  | flooded | POSTPOST | PRE | 8 | 8 | 35 | 0.016 | 0.047 | * |
| ***nirK*** | flooded | EXP | PRE | 8 | 8 | 0 | 0.008 | 0.016 | * |
|  | flooded | POST | PRE | 8 | 8 | 0 | 0.008 | 0.016 | * |
|  | flooded | POSTPOST | PRE | 8 | 8 | 0 | 0.008 | 0.016 | * |
| ***nirS*** | flooded | EXP | POSTPOST | 8 | 8 | 2 | 0.023 | 0.035 | * |
|  | flooded | EXP | PRE | 8 | 8 | 0 | 0.008 | 0.016 | * |
|  | flooded | POST | POSTPOST | 8 | 8 | 0 | 0.008 | 0.016 | * |
|  | flooded | POST | PRE | 8 | 8 | 0 | 0.008 | 0.016 | * |
| ***nosZI*** | flooded | EXP | POST | 8 | 8 | 0 | 0.008 | 0.016 | * |
|  | flooded | EXP | POSTPOST | 8 | 8 | 35 | 0.016 | 0.023 | * |
|  | flooded | EXP | PRE | 8 | 8 | 33 | 0.039 | 0.047 | * |
|  | flooded | POST | POSTPOST | 8 | 8 | 36 | 0.008 | 0.016 | * |
|  | flooded | POST | PRE | 8 | 8 | 36 | 0.008 | 0.016 | * |
| ***nosZII*** | flooded | EXP | POST | 8 | 8 | 35 | 0.016 | 0.023 | * |
|  | flooded | POST | POSTPOST | 8 | 8 | 0 | 0.008 | 0.016 | * |
|  | flooded | POST | PRE | 8 | 8 | 0 | 0.008 | 0.016 | * |
|  | flooded | POSTPOST | PRE | 8 | 8 | 36 | 0.008 | 0.016 | * |
| ***nrfA*** | flooded | POST | PRE | 8 | 8 | 0 | 0.008 | 0.047 | * |
| **Fungal *nirK*** | flooded | EXP | POSTPOST | 8 | 8 | 0 | 0.008 | 0.016 | * |
|  | flooded | POST | POSTPOST | 8 | 8 | 0 | 0.008 | 0.016 | * |
|  | flooded | POSTPOST | PRE | 8 | 8 | 36 | 0.008 | 0.016 | * |

**Supplementary Table 4:** Significant differences in marker gene abundances (qPCR) between control and flooded plots in different experimental periods. Abbreviations: pre-flood period (PRE), flooding (EXP), post-flood period (POST), a year after the flooding (POSTPOST).

| **Var1** | **Var2** | **p-value** | **Gene** | **Treatment** |
| --- | --- | --- | --- | --- |
| POST | EXP | <0.05 | Bacterial 16S rRNA | control |
| POSTPOST | POST | <0.05 | Bacterial 16S rRNA | control |
| PRE | POST | <0.05 | Bacterial 16S rRNA | control |
| PRE | POSTPOST | <0.05 | Bacterial 16S rRNA | control |
| POST | EXP | <0.05 | Bacterial 16S rRNA | flooded |
| POSTPOST | POST | <0.05 | Bacterial 16S rRNA | flooded |
| POST | EXP | <0.05 | Bacterial *amoA* | flooded |
| POSTPOST | EXP | <0.05 | Bacterial *amoA* | flooded |
| PRE | EXP | <0.05 | Bacterial *amoA* | flooded |
| PRE | POST | <0.01 | COMAMMOX *amoA* | control |
| POST | EXP | <0.001 | COMAMMOX *amoA* | flooded |
| PRE | EXP | <0.05 | COMAMMOX amoA | flooded |
| POSTPOST | POST | <0.001 | COMAMMOX *amoA* | flooded |
| PRE | POST | <0.001 | COMAMMOX *amoA* | flooded |
| POSTPOST | EXP | <0.01 | n-damo 16S rRNA | flooded |
| POSTPOST | POST | <0.01 | n-damo 16S rRNA | flooded |
| PRE | POSTPOST | <0.001 | n-damo 16S rRNA | flooded |
| POST | EXP | <0.05 | *nifH* | control |
| POSTPOST | EXP | <0.05 | *nifH* | control |
| POSTPOST | POST | <0.05 | *nifH* | control |
| PRE | POSTPOST | <0.05 | *nifH* | control |
| PRE | POSTPOST | <0.05 | *nifH* | flooded |
| PRE | EXP | <0.01 | *nirK* | control |
| PRE | POST | <0.01 | *nirK* | control |
| PRE | POSTPOST | <0.05 | *nirK* | control |
| PRE | EXP | <0.001 | *nirK* | flooded |
| PRE | POST | <0.001 | *nirK* | flooded |
| PRE | POSTPOST | <0.01 | *nirK* | flooded |
| POST | EXP | <0.01 | *nosZI* | flooded |
| POSTPOST | POST | <0.01 | *nosZI* | flooded |
| PRE | POST | <0.01 | *nosZI* | flooded |
| POSTPOST | POST | <0.05 | *nosZII* | flooded |
| PRE | POST | <0.01 | *nrfA* | control |
| PRE | POST | <0.001 | *nrfA* | flooded |
| POSTPOST | EXP | <0.01 | Fungal *nirK* | flooded |
| POSTPOST | POST | <0.01 | Fungal *nirK* | flooded |
| PRE | POSTPOST | <0.05 | Fungal *nirK* | flooded |
| POSTPOST | EXP | <0.05 | *pmoA* | flooded |
| PRE | POSTPOST | <0.05 | *pmoA* | flooded |

**Supplementary Table 5:** Significant changes fungal genus (top 50) relative abundance in the CP and FP. Statistically significant differences between periods (PRE vs. EXP, EXP vs. POST, POST vs. POSTPOST) were determined using the Wilcoxon rank-sum test. Asterisks (*) indicate Benjamini-Hochberg corrected (p < 0.05) statistical significance. Abbreviations: pre-flood period (PRE), flooding (EXP), post-flood period (POST), a year after the flooding (POSTPOST). Significant differences (p<0.05) are shown in red.

|  | **The Wilcoxon rank-sum test p-values** | | | | | |
| --- | --- | --- | --- | --- | --- | --- |
|  | **Flooded plot** | | | **Control plot** | | |
| Genus | **PRE vs EXP** | **EXP vs POST** | **POST vs POSTPOST** | **PRE vs EXP** | **EXP vs POST** | **POST vs POSTPOST** |
| *Amphisphaeriaceae* | 0.743 | 0.855 | 0.018 | 0.171 | 0.488 | 0.602 |
| *Apiotrichum* | 0.126 | 0.181 | 0.118 | 0.270 | 0.189 | 0.149 |
| *Ascobolaceae* | 0.087 | 0.831 | 0.115 | 0.701 | 0.452 | 0.636 |
| *Ascobolus* | 0.985 | 0.955 | 0.713 | 0.372 | 0.318 | 0.671 |
| *Cercomonadida* | 0.001* | 0.040 | 0.257 | 0.007 | 0.713 | 0.799 |
| *Cercozoa* | 0.094 | 0.534 | 0.561 | 0.052 | 0.189 | 0.932 |
| *Chytridiomycota* | 0.109 | 0.010 | 0.118 | 0.003 | 0.003 | 0.075 |
| *Coprinellus* | 0.040* | 0.376 | 0.736 | 0.083 | 0.564 | 0.051 |
| *Cortinarius* | 0.181 | 0.376 | 0.343 | 0.083 | 0.318 | 0.552 |
| *Eocronartium* | 0.277 | 0.198 | 0.657 | 0.634 | 0.711 | 0.931 |
| *Ganoderma* | 0.187 | 0.522 | 0.561 | 0.431 | 1.000 | 0.552 |
| *GS02* | 0.009 | 0.066 | 0.618 | 0.066 | 0.083 | 1.000 |
| *GS07* | 0.318 | 0.637 | 0.927 | 0.793 | 0.875 | 0.799 |
| *GS08* | 0.300 | 1.000 | 0.783 | 0.637 | 0.793 | 0.149 |
| *Hebelomataceae* | 0.510 | 0.396 | 0.004 | 0.227 | 0.128 | 0.203 |
| *Ichthyosporea* | 0.300 | 0.337 | 0.312 | 0.637 | 0.637 | 0.445 |
| *Idriella* | 0.376 | 0.337 | 0.284 | 0.875 | 0.564 | 0.552 |
| *Inocybe* | 0.924 | 1.000 | 0.828 | 0.072 | 0.793 | 0.270 |
| *Lactarius* | 0.235 | 0.734 | 0.878 | 1.000 | 0.066 | 0.350 |
| *Limnoperdaceae* | 0.984 | 0.724 | 0.057 | 0.050 | 0.561 | 0.549 |
| *Linnemannia* | 0.895 | 0.611 | 0.035 | 1.000 | 1.000 | 0.799 |
| *Melampsoridium* | 0.007 | 0.572 | 0.603 | 0.007 | 0.066 | 0.075 |
| *Melanogaster* | 0.158 | 0.777 | 0.830 | 0.083 | 0.372 | 0.552 |
| *Microbotryales* | 0.523 | 0.575 | 0.680 | 0.832 | 0.712 | 0.269 |
| *Minimedusa* | 0.087 | 0.063 | 0.362 | 0.325 | 0.450 | 0.792 |
| *Mortierella* | 0.585 | 0.462 | 0.257 | 0.270 | 0.713 | 0.799 |
| *Mycena* | 0.985 | 0.479 | 0.876 | 0.685 | 0.685 | 0.130 |
| *Naucoria* | 0.266 | 0.418 | 0.691 | 0.875 | 0.958 | 0.671 |
| Genus | **The Wilcoxon rank-sum test p-values** | | | | | |
|  | **Flooded plot** | | | **Control plot** | | |
|  | **PRE vs EXP** | **EXP vs POST** | **POST vs POSTPOST** | **PRE vs EXP** | **EXP vs POST** | **POST vs POSTPOST** |
| *Oliveonia* | 0.775 | 0.038 | 0.492 | 1.000 | 0.916 | 0.266 |
| *Operculomyces* | 0.910 | 0.520 | 0.736 | 0.875 | 0.031 | 0.496 |
| *Paxillus* | 0.266 | 1.000 | 0.481 | 0.024 | 0.958 | 0.350 |
| *Peziza* | 0.576 | 0.576 | 0.702 |  |  |  |
| *Pleotrichocladium* | 0.510 | 0.200 | 0.603 | 0.431 | 0.713 | 0.051 |
| *Podila* | 0.101 | 0.486 | 0.736 | 0.104 | 0.227 | 0.350 |
| *Protomyces* | 0.849 | 0.375 | 0.193 | 0.072 | 0.115 | 1.000 |
| *Pseudosigmoidea* | 0.308 | 0.545 | 0.081 | 0.400 | 0.290 | 0.549 |
| *Rhizophydiaceae* | 0.638 | 0.692 | 0.520 | 0.372 | 0.227 | 0.671 |
| *Rhizophydiales* | 0.925 | 0.169 | 0.444 | 0.713 | 0.128 | 0.350 |
| *Rhizophydium* | 0.318 | 0.749 | 0.054 | 0.318 | 0.564 | 0.552 |
| *Rigidoporus* | 0.349 |  | 0.185 | 0.750 | 1.000 | 0.840 |
| *Russula* | 0.414 | 0.482 | 0.877 | 0.400 | 0.674 | 0.610 |
| *Saccharomycetales* | 0.598 | 0.534 | 0.168 | 0.793 | 0.958 | 0.270 |
| *Saitozyma* | 0.636 | 0.820 | 0.733 | 0.709 | 0.341 | 0.120 |
| *Solicoccozyma* | 0.250 | 0.118 | 0.284 | 0.495 | 0.793 | 0.671 |
| *Spirosphaera* | 0.865 | 0.158 | 0.520 | 0.052 | 0.128 | 0.799 |
| *Tetracladium* | 0.187 | 0.090 | 0.188 | 0.958 | 0.270 | 1.000 |
| *Thelephora* | 0.777 | 0.692 | 0.312 | 0.875 | 0.637 | 0.552 |
| *Tomentella* | 0.720 | 0.376 | 0.561 | 0.431 | 0.564 | 0.799 |
| *Trichosporiella* | 0.100 | 0.288 | 0.687 | 0.793 | 0.564 | 0.203 |
| Umbelopsis | 0.366 | 0.783 | 0.949 | 0.832 | 0.269 | 0.931 |

**Supplementary Table 6**. Bacterial Permutation test for adonis under reduced model. Number of permutations: 999.

|  | Df | SumOfSqs | R2 | F | Pr(>F) |  |
| --- | --- | --- | --- | --- | --- | --- |
| **Treatment** | 1 | 1.1427 | 0.05881 | 5.1812 | 0.001 | ******* |
| **Experiment** | 3 | 1.0126 | 0.05211 | 1.5304 | 0.019 | ***** |
| **Treatment:Experiment** | 3 | 0.5142 | 0.02646 | 0.7771 | 0.915 |  |
| **Residual** | 76 | 16.7623 | 0.86262 |  |  |  |
| **Total** | 83 | 19.4318 | 1 |  |  |  |

Signif. codes: 0 ‘***’ 0.001 ‘**’ 0.01 ‘*’ 0.05 ‘.’ 0.1 ‘ ’ 1

**Supplementary Table 7.** Fungal Permutation test for adonis under reduced model. Number of permutations: 999.

|  | Df | SumOfSqs | R2 | F | Pr(>F) |  |
| --- | --- | --- | --- | --- | --- | --- |
| **Treatment** | 1 | 0.3543 | 0.01581 | 1.3503 | 0.071 | . |
| **Experiment** | 3 | 0.7504 | 0.03349 | 0.9534 | 0.588 |  |
| **Treatment:Experiment** | 3 | 1.3646 | 0.06089 | 1.7336 | 0.001 | *** |
| **Residual** | 76 | 19.9405 | 0.88981 |  |  |  |
| **Total** | 83 | 22.4098 | 1 |  |  |  |

Signif. codes: 0 ‘***’ 0.001 ‘**’ 0.01 ‘*’ 0.05 ‘.’ 0.1 ‘ ’ 1

**Supplementary Table 8.** AMF Permutation test for adonis under reduced model. Number of permutations: 999.

|  | Df | SumOfSqs | R2 | F | Pr(>F) |  |
| --- | --- | --- | --- | --- | --- | --- |
| **Treatment** | 1 | 1.6004 | 0.06875 | 6.4503 | 0.001 | *** |
| **Experiment** | 3 | 2.0394 | 0.0876 | 2.7399 | 0.001 | *** |
| **Treatment:Experiment** | 3 | 0.7833 | 0.03365 | 1.0523 | 0.381 |  |
| **Residual** | 76 | 18.8568 | 0.81 |  |  |  |
| **Total** | 83 | 23.28 | 1 |  |  |  |

Signif. codes: 0 ‘***’ 0.001 ‘**’ 0.01 ‘*’ 0.05 ‘.’ 0.1 ‘ ’ 1

**Supplementary Table 9.** The mean and standard error of original reads of bacteria, fungi and AMF for flooded (plot ID 1-8 shown with grey lines) and control plots (plot ID 9-12).

|  |  |  | **Bacteria** | | **Fungi** | | **AMF** | |
| --- | --- | --- | --- | --- | --- | --- | --- | --- |
| **Sampling campaign** | **Treatment** | **Experiment** | **mean** | **standard error** | **mean** | **standard error** | **mean** | **standard error** |
| 1 | Control | PRE | 100792 | 2234.073 | 165040 | 5241.51 | 38565 | 32055.182 |
|  | Flooded | PRE | 89480 | 3694.014 | 141110 | 11530.36 | 10229 | 3729.532 |
| 2 | Control | PRE | 78821 | 1661.952 | 142907 | 926.09 | 30894 | 15112.109 |
|  | Flooded | PRE | 73531 | 2776.757 | 118577 | 9998.77 | 7446 | 2351.905 |
| 3 | Control | EXP | 54879 | 12010.501 | 155387 | 1326.66 | 17887 | 14720.355 |
|  | Flooded | EXP | 63810 | 6165.435 | 129428 | 7570.20 | 19339 | 11611.970 |
| 4 | Control | EXP | 71689 | 1961.964 | 129630 | 66743.90 | 75706 | 66743.904 |
|  | Flooded | EXP | 58682 | 8807.112 | 113508 | 5338.97 | 12457 | 6028.196 |
| 5 | Control | POST | 105201 | 3352.217 | 124892 | 4544.74 | 4184 | 532.514 |
|  | Flooded | POST | 87738 | 3584.729 | 99242 | 10168.03 | 36896 | 29897.516 |
| 6 | Control | POST | 71844 | 10691.604 | 138842 | 9650.77 | 7682 | 4023.992 |
|  | Flooded | POST | 74490 | 3707.716 | 114207 | 6009.24 | 32253 | 15305.271 |
| 7 | Control | POSTPOST | 81198 | 1277.758 | 123787 | 7363.17 | 10988 | 5351.158 |
|  | Flooded | POSTPOST | 80730 | 3676.357 | 112131 | 7138.95 | 13981 | 7910.285 |


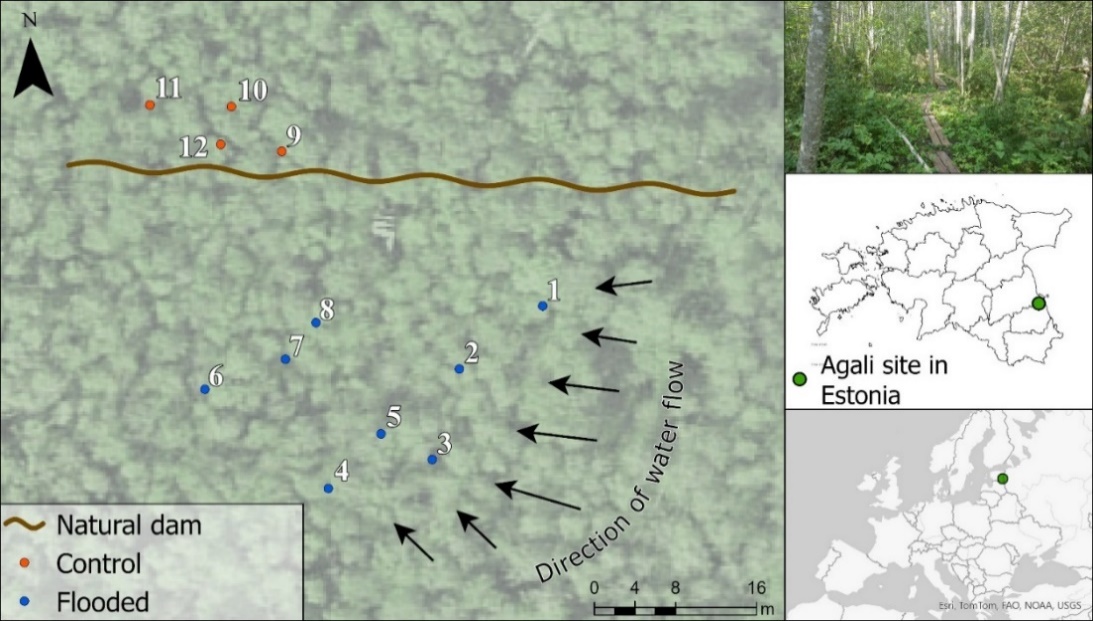


**Supplementary Figure 1.** Agali sampling area in Estonia. Blue points indicate the flooded area (points 1–8), and orange points indicate the control area (points 9–12). A brown curved line represents a one-metre natural dyke separating the two sampling areas. The orthophoto is from 2017, provided by the Estonian Land- and Spatial Development Board. European map is Esri’s Light Gray Canvas (2025) basemap and Estonian provinces map is from Estonian Land- and Spatial Development Board (2025).


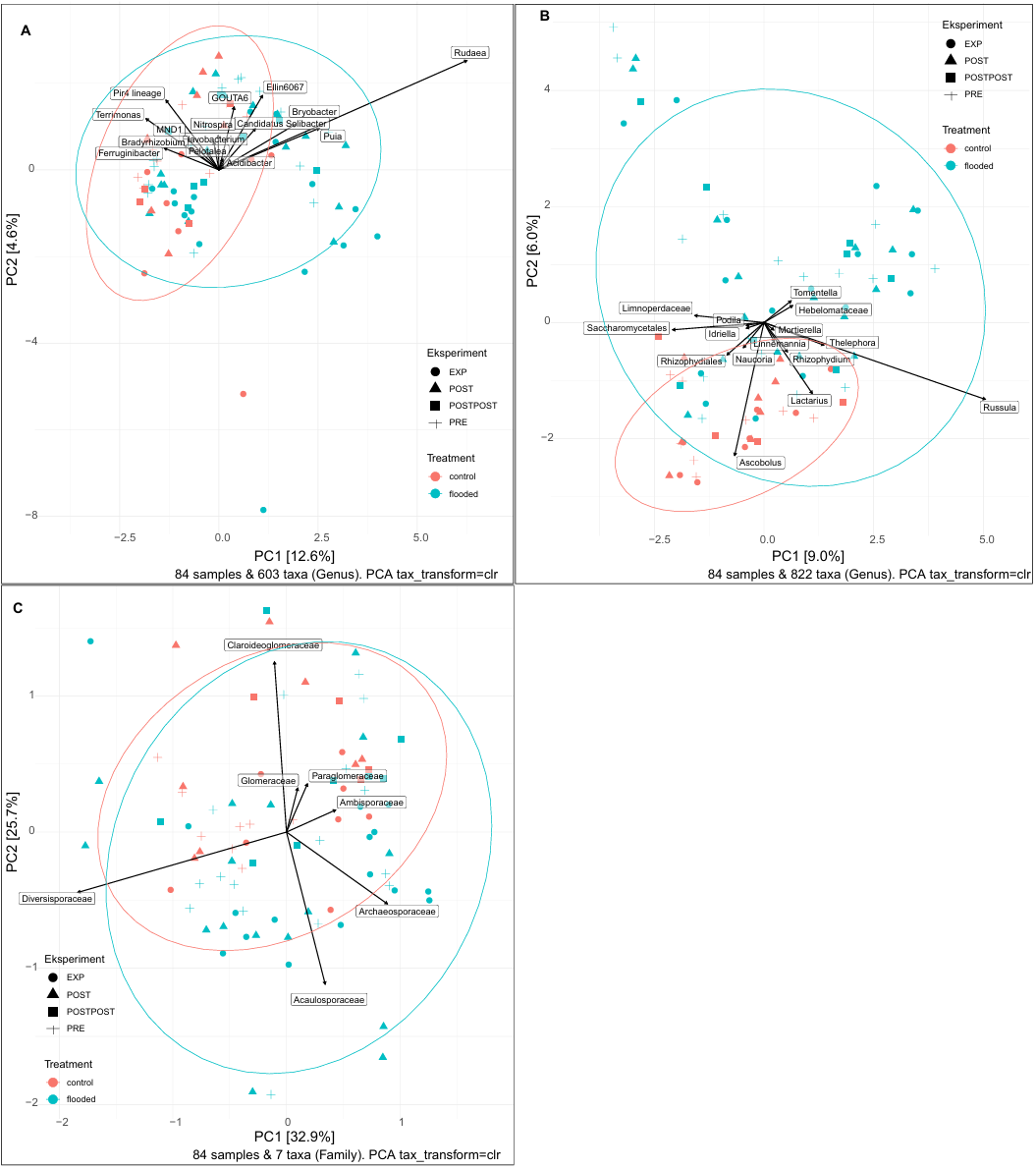


**Supplementary Figure 2**. Principal Components Analysis (PCA) ordination plots showing relationships of between bacterial (A) genera, fungal (B) genera and AMF (C) family. Ellipses show confidence level 0.95. Red colour represents control plot and blue colour flooded plot. Abbreviations: pre-flood period (PRE), flooding period (EXP), post-flood period (POST), a year after the flooding (POSTPOST).


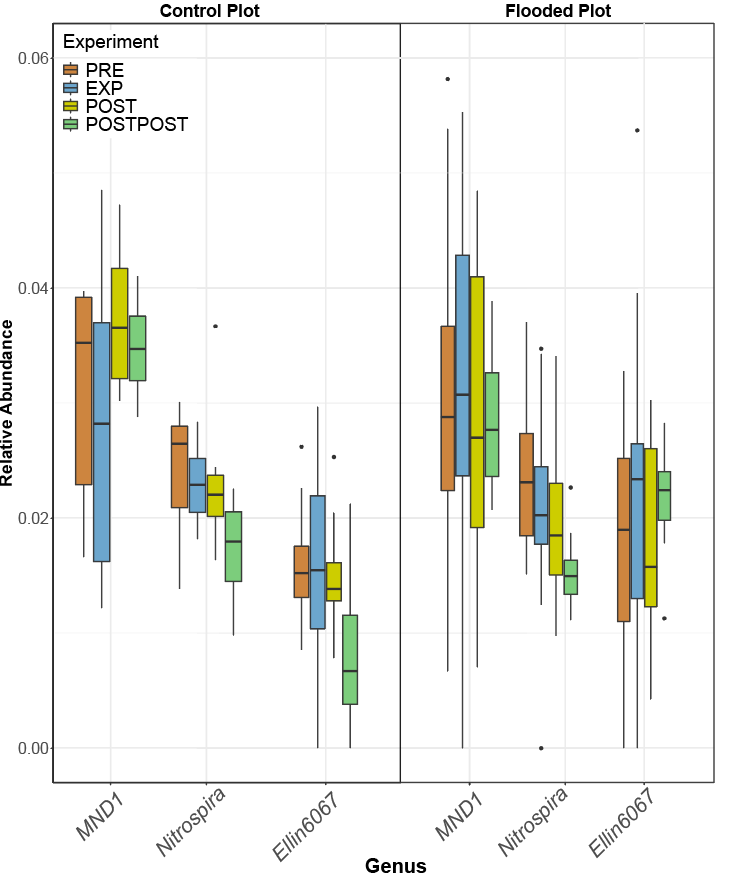


**Supplementary Figure 3:** Changes in relative abundance of *MND1*, *Nitrospira* and *Ellin6067* in control and flooded plot. Abbreviations: pre-flood period (PRE), flooding period (EXP), post-flood period (POST), a year after the flooding (POSTPOST).


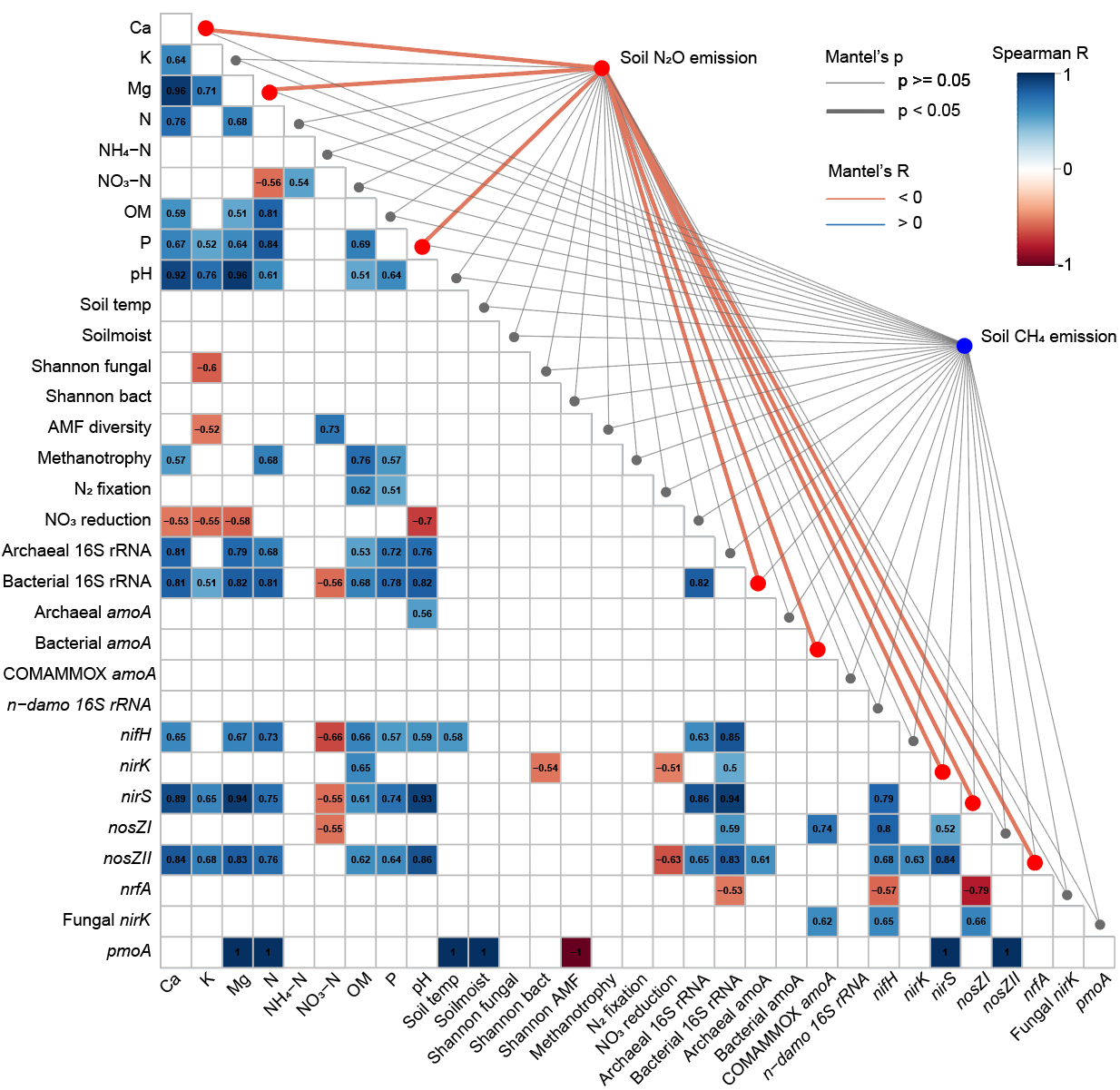


**Supplementary Figure 4**. Spearman correlation and Mantel test before the flooding (PRE) period in FP. The correlation table displays only significant (p<0.05) correlations between environmental variables, marker genes, processes, and diversity. Mantel test results show soil N_2_O (red) and CH_4_ (blue) emissions in relation to environmental variables, marker genes, processes and diversity, represented by lines. Thick lines indicate significant relationships (p<0.05), with colour denoting positive (blue line) or negative (red line) relationships.


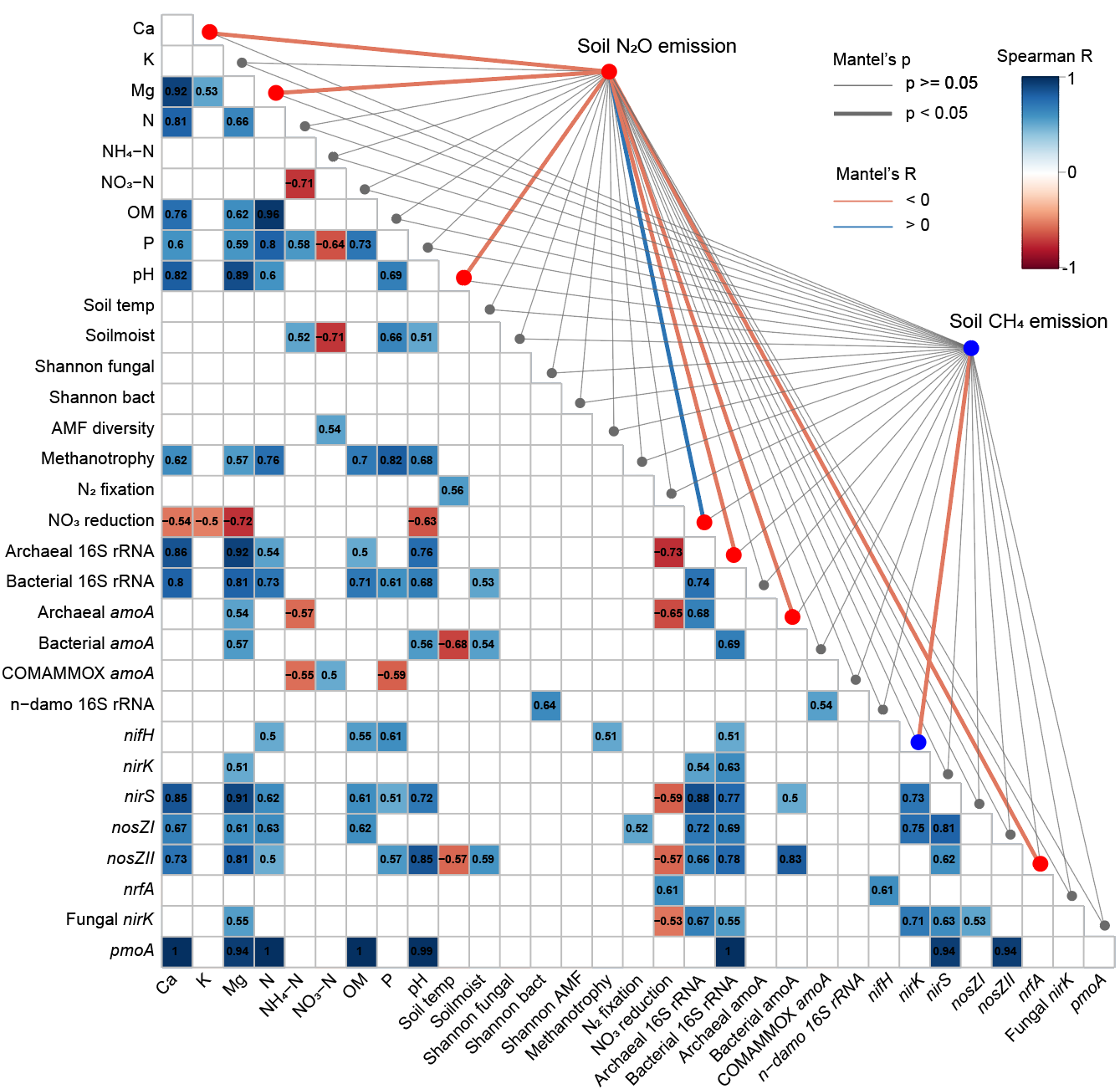


**Supplementary Figure 5.** Spearman correlation and Mantel test after the flooding (POST) period in the FP. The correlation table displays only significant (p<0.05) correlations between environmental variables, marker genes, processes, and diversity. Mantel test results show soil N_2_O (red) and CH_4_ (blue) emissions in relation to environmental variables, marker genes, processes and diversity, represented by lines. Thick lines indicate significant relationships (p<0.05), with colour denoting positive (blue line) or negative (red line) relationships.


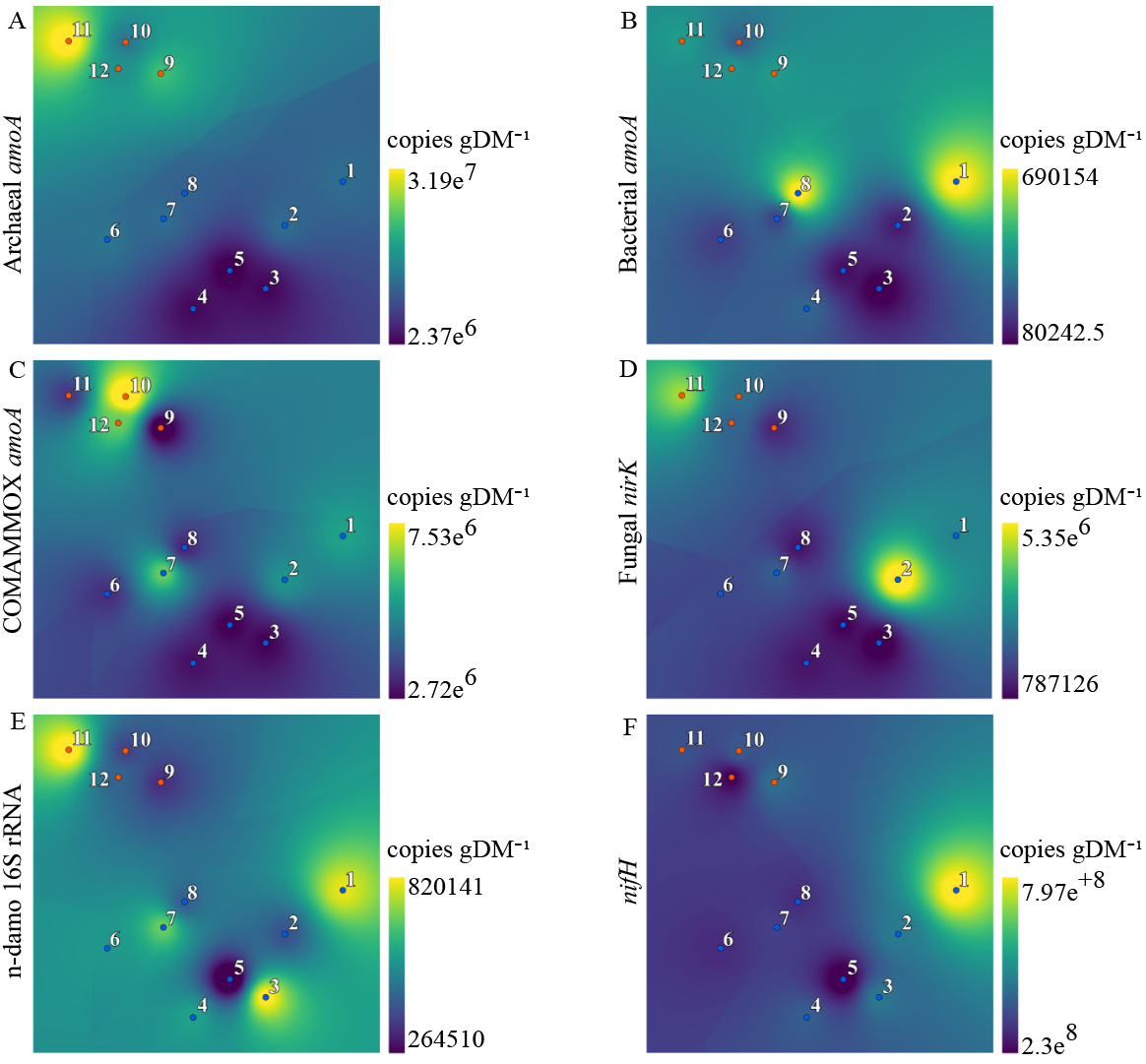


**Supplementary Figure 6.** Sampling area archaeal *amoA* (A), bacterial *amoA* (B), COMAMMOX *amoA* (C), fungal *nirK* (D), n-damo 16S rRNA (E) and *nifH* (F) abundances during the EXP period. CP sampling points are shown in red, and FP sampling points are in blue. Absolute abundances (gene copies per gram of dry matter (copies gDM^−1^)) from quantitative PCR were used for interpolation.


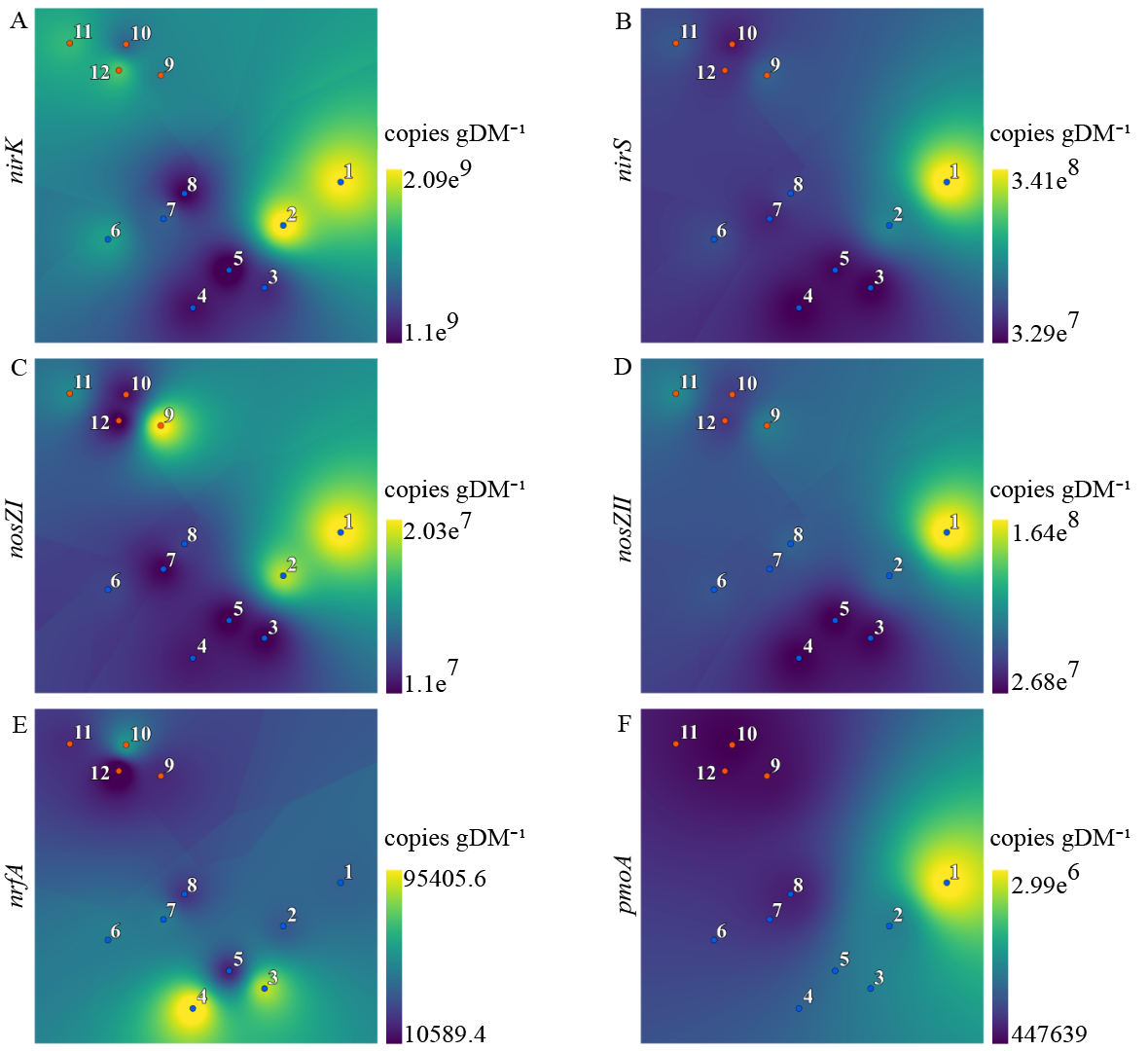


**Supplementary Figure 7.** Sampling area *nirK* (A), *nirS* (B), *nosZI* (C), *nosZII* (D), *nrfA* (E) and *pmoA* (F) abundances during the EXP period. CP sampling points are shown in red, and FP sampling points are in blue. Absolute abundances (gene copies per gram of dry matter (copies gDM^−1^)) from quantitative PCR were used for interpolation.


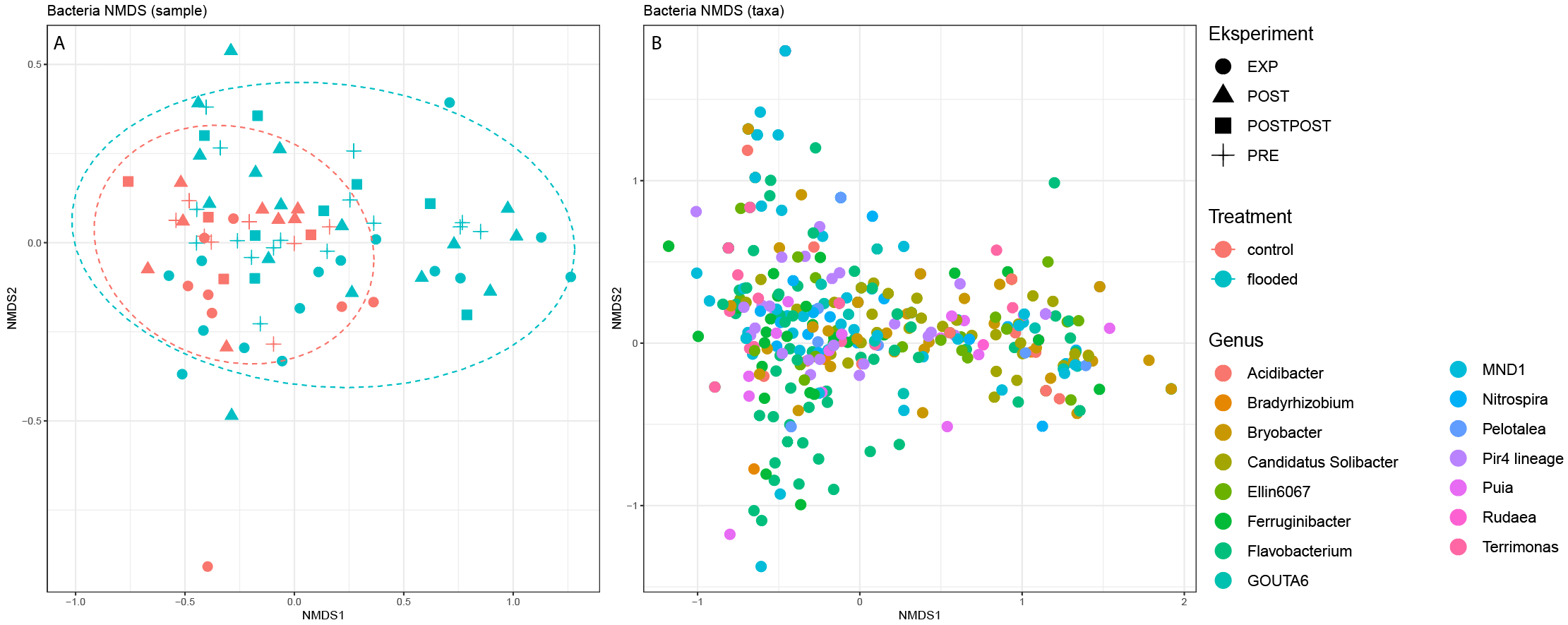


**Supplementary Figure 8.** Nonmetric Multidimensional Scaling (NMDS) ordination of bacterial communities based on Bray–Curtis dissimilarity. In plot A each point represents one sample, coloured by treatment (PRE, EXP, POST, POSTPOST) and shaped by experimental group. Ellipses indicate 95% confidence intervals around treatment group centroids. Plot B shows the distribution of the 15 most abundant bacterial genera across samples, based on Bray–Curtis dissimilarity. Points are coloured by genus.


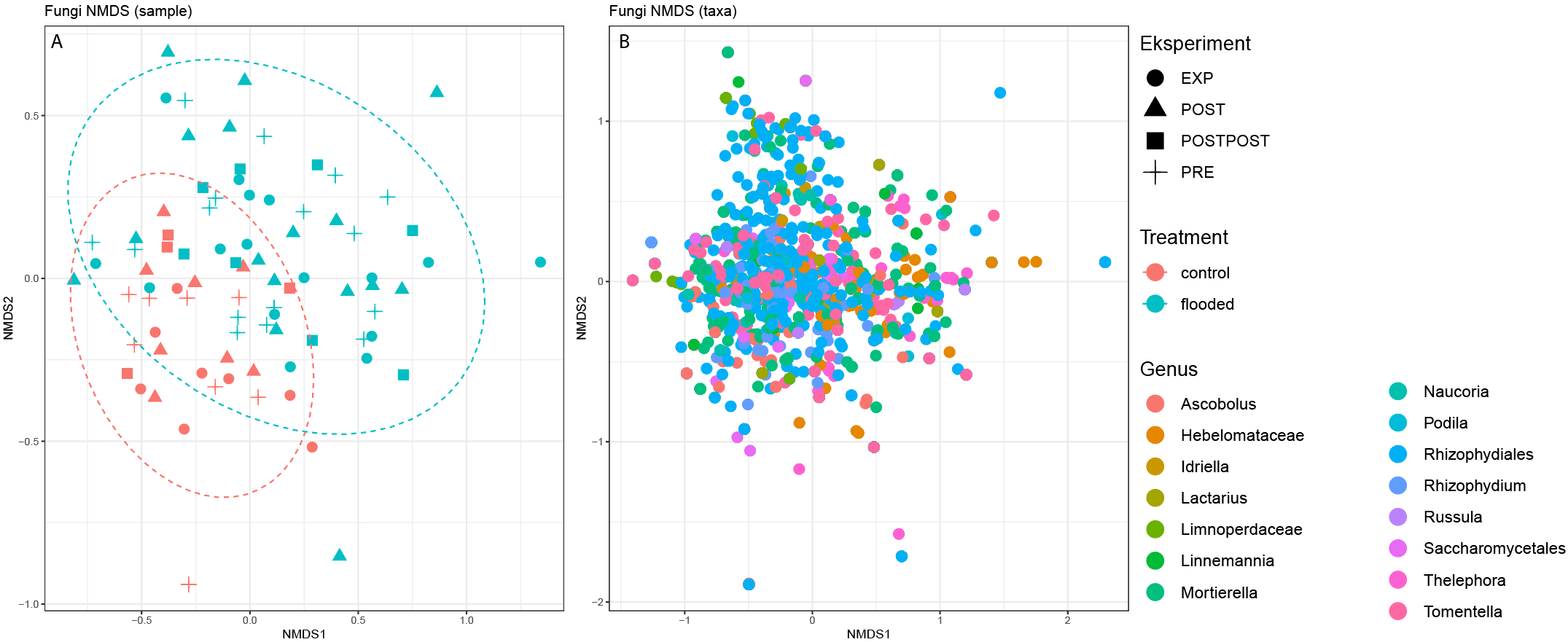


**Supplementary Figure 9.** Nonmetric Multidimensional Scaling (NMDS) ordination of fungal communities based on Bray–Curtis dissimilarity. In plot A each point represents one sample, coloured by treatment (PRE, EXP, POST, POSTPOST) and shaped by experimental group. Ellipses indicate 95% confidence intervals around treatment group centroids. Plot B shows the distribution of the 15 most abundant fungal genera across samples, based on Bray–Curtis dissimilarity. Points are coloured by genus.


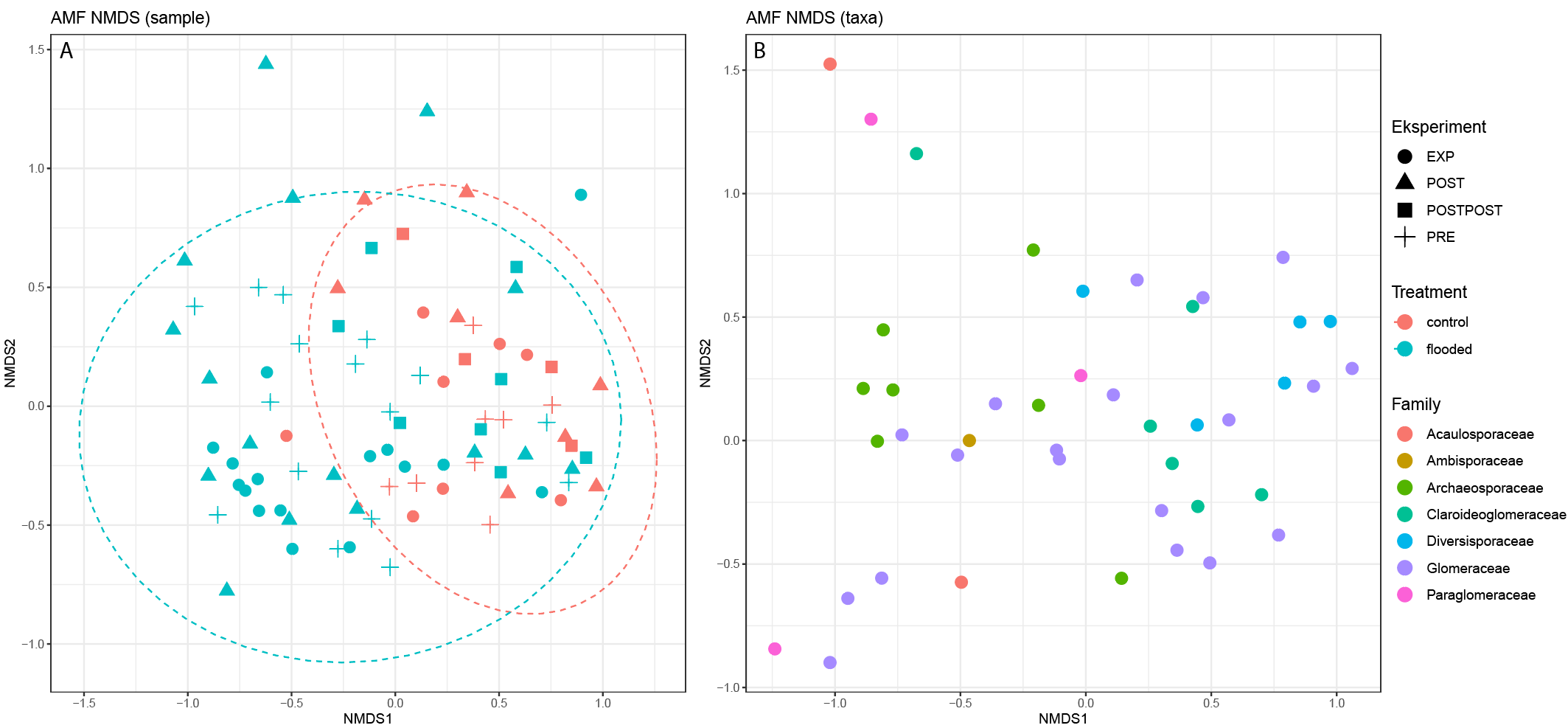


**Supplementary Figure 10.** Nonmetric Multidimensional Scaling (NMDS) ordination of AMF communities based on Bray–Curtis dissimilarity. In plot A each point represents one sample, coloured by treatment (PRE, EXP, POST, POSTPOST) and shaped by experimental group. Ellipses indicate 95% confidence intervals around treatment group centroids. Plot B shows the distribution of the AMF families across samples, based on Bray–Curtis dissimilarity. Points are coloured by family.


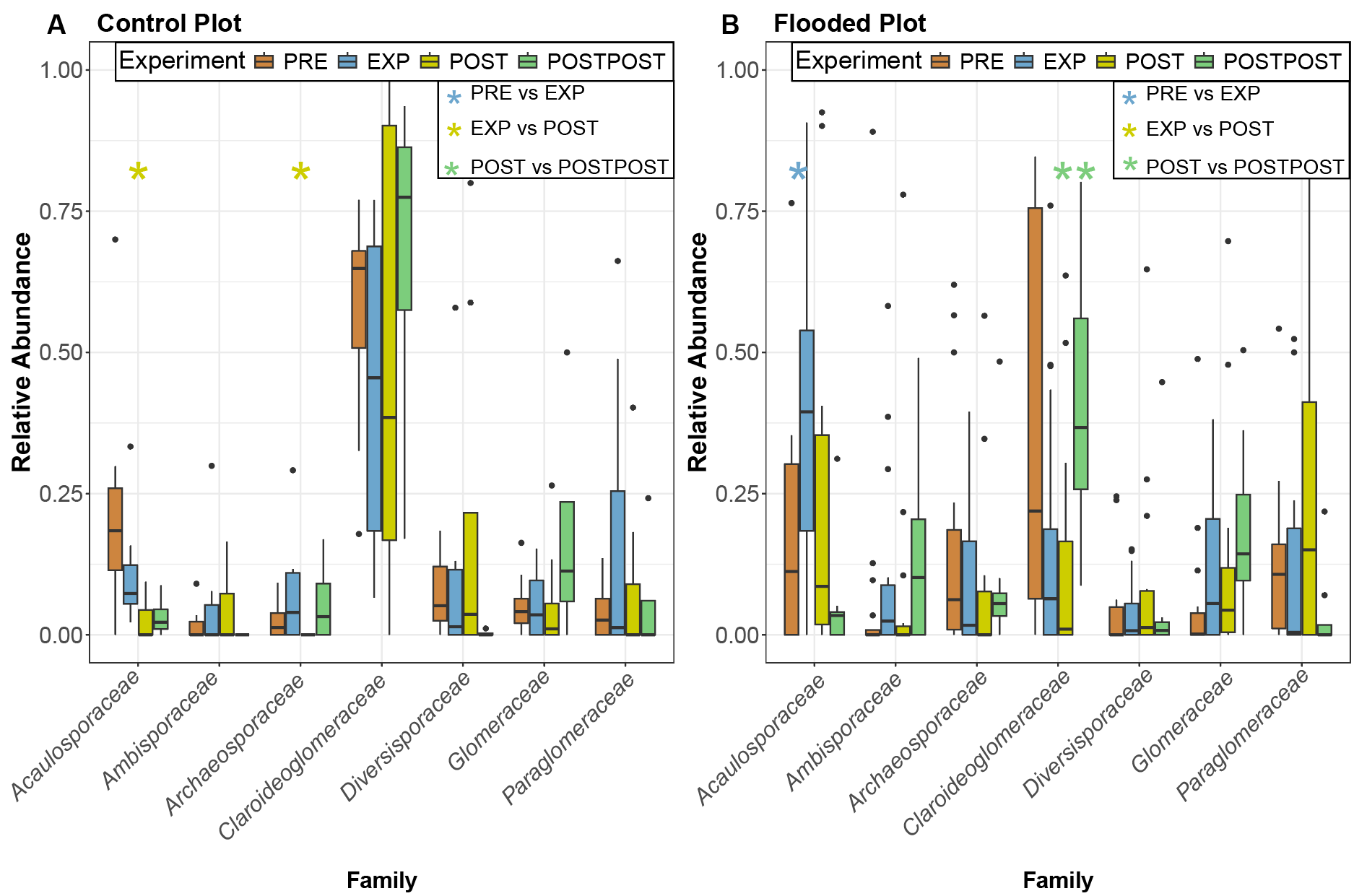


**Supplementary 11:** Relative abundances of arbuscular mycorrhizal fungi (AMF) at the family (A) level in the control plots (CP) and flooded plots (FP) during different experiment periods, based on fungal sequencing data (ITS). The Wilcoxon rank-sum test determined statistically significant differences between periods (PRE vs EXP, EXP vs POST, POST vs POSTPOST). Asterisks (*) indicate statistical significance (* – p < 0.05, ** – p < 0.01, *** – p < 0.001), with blue, yellow, and green denoting changes between PRE-EXP, EXP-POST, and POST-POSTPOST, respectively. Abbreviations: pre-flood period (PRE), flooding period (EXP), post-flood period (POST), a year after the flooding (POSTPOST).

**Supplementary reference**

Chen, H., Yu, F., & Shi, W. (2016). Detection of N2O-producing fungi in environment using nitrite reductase gene (nirK)-targeting primers. *Fungal Biology*, *120*(12), 1479–1492.

Costello, A. M., & Lidstrom, M. E. (1999). Molecular characterization of functional and phylogenetic genes from natural populations of methanotrophs in lake sediments. *Applied and Environmental Microbiology*, *65*(11), 5066–5074.

Dethlefsen, L., Huse, S., Sogin, M. L., & Relman, D. A. (2008). The pervasive effects of an antibiotic on the human gut microbiota, as revealed by deep 16S rRNA sequencing. *PLoS Biology*, *6*(11), e280.

Espenberg, M., Truu, M., Truu, J., Maddison, M., Nõlvak, H., Järveoja, J., & Mander, Ü. (2016). Impact of reed canary grass cultivation and mineral fertilisation on the microbial abundance and genetic potential for methane production in residual peat of an abandoned peat extraction area. *PloS One*, *11*(9), e0163864.

Esri. (2025). *Light Gray Canvas*. https://www.arcgis.com/home/item.html?id=979c6cc89af9449cbeb5342a439c6a76

Ettwig, K. F., van Alen, T., van de Pas-Schoonen, K. T., Jetten, M. S., & Strous, M. (2009). Enrichment and molecular detection of denitrifying methanotrophic bacteria of the NC10 phylum. *Applied and Environmental Microbiology*, *75*(11), 3656–3662.

Hallin, S., & Lindgren, P.-E. (1999). PCR detection of genes encoding nitrite reductase in denitrifying bacteria. *Applied and Environmental Microbiology*, *65*(4), 1652–1657.

Henry, S., Bru, D., Stres, B., Hallet, S., & Philippot, L. (2006). Quantitative detection of the nosZ gene, encoding nitrous oxide reductase, and comparison of the abundances of 16S rRNA, narG, nirK, and nosZ genes in soils. *Applied and Environmental Microbiology*, *72*(8), 5181–5189.

Jones, C. M., Graf, D. R., Bru, D., Philippot, L., & Hallin, S. (2013). The unaccounted yet abundant nitrous oxide-reducing microbial community: A potential nitrous oxide sink. *The ISME Journal*, *7*(2), 417–426.

Kandeler, E., Deiglmayr, K., Tscherko, D., Bru, D., & Philippot, L. (2006). Abundance of narG, nirS, nirK, and nosZ genes of denitrifying bacteria during primary successions of a glacier foreland. *Applied and Environmental Microbiology*, *72*(9), 5957–5962.

Land and Spatial Development Board. (2017). *Orthophoto Metadata by Year*. Spatial Data. https://geoportaal.maaamet.ee/eng/spatial-data/orthophotos/orthophoto-metadata-by-year-p350.html

Land and Spatial Development Board. (2025). *Administrative and Settlement Division*. Spatial Data. https://geoportaal.maaamet.ee/eng/spatial-data/administrative-and-settlement-division-p312.html

Liu, Z., Lozupone, C., Hamady, M., Bushman, F. D., & Knight, R. (2007). Short pyrosequencing reads suffice for accurate microbial community analysis. *Nucleic Acids Research*, *35*(18), e120.

Rotthauwe, J.-H., Witzel, K.-P., & Liesack, W. (1997). The ammonia monooxygenase structural gene amoA as a functional marker: Molecular fine-scale analysis of natural ammonia-oxidizing populations. *Applied and Environmental Microbiology*, *63*(12), 4704–4712.

Takeuchi, J. (2006). Habitat segregation of a functional gene encoding nitrate ammonification in estuarine sediments. *Geomicrobiology Journal*, *23*(2), 75–87.

Tourna, M., Freitag, T. E., Nicol, G. W., & Prosser, J. I. (2008). Growth, activity and temperature responses of ammonia-oxidizing archaea and bacteria in soil microcosms. *Environmental Microbiology*, *10*(5), 1357–1364.

Ueda, T., Suga, Y., Yahiro, N., & Matsuguchi, T. (1995). Remarkable N2-fixing bacterial diversity detected in rice roots by molecular evolutionary analysis of nifH gene sequences. *Journal of Bacteriology*, *177*(5), 1414–1417.

Wang, M., Huang, G., Zhao, Z., Dang, C., Liu, W., & Zheng, M. (2018). Newly designed primer pair revealed dominant and diverse comammox amoA gene in full-scale wastewater treatment plants. *Bioresource Technology*, *270*, 580–587.
